# Sex–specific associations between frailty and long-term outcomes in patients with acute myocardial infarction: a national population-based study

**DOI:** 10.1016/j.lanepe.2026.101612

**Published:** 2026-02-12

**Authors:** Hasan Mohiaddin, Chijioke Horatio Mosanya, Claire Lawson, Kamlesh Khunti, Angela Wood, Iain B. Squire, Gerry P. McCann, Abdulla A. Damluji, Sergio Buccheri, Mohamad A. Alkhouli, Mamas A. Mamas, Muhammad Rashid

**Affiliations:** a National Institute for Health Research Leicester Biomedical Research Centre and British Heart Foundation Centre of Research Excellence, Leicester, UK; b Keele Cardiovascular Research Group, Centre for Prognosis Research, School of Medicine, Keele University, UK; c Cardiology Department, Glenfield Hospital, University Hospitals of Leicester NHS Foundation Trust, UK; d Leicester Real World Evidence Unit, Diabetes Research Centre, University of Leicester, Leicester, LE5 4PW, UK; e NIHR Applied Research Collaborations East Midlands, University Hospitals of Leicester NHS Trust, Leicester, UK; f Cardiovascular Epidemiology Unit, Department of Public Health and Primary Care, University of Cambridge, Cambridge, United Kingdom of Great Britain and Northern Ireland; g The Cardiovascular Center on Aging, Department of Cardiovascular Medicine, the Cleveland Clinic Foundation, Cleveland, OH, USA; h Department of Medical Sciences, Cardiology and Uppsala Clinical Research Center, Uppsala University, Uppsala, Sweden; i Department of Cardiology, Mayo Clinic School of Medicine, Rochester, MN, USA

**Keywords:** Frailty, Acute myocardial infarction

## Abstract

**Background:**

Frailty and female sex are both recognised independent predictors of adverse outcomes after acute myocardial infarction (AMI). While females presenting with AMI are known to have a higher burden of frailty than males, it is unknown whether this fully explains sex-based disparities in outcomes, or if the prognostic impact of frailty itself differs between the sexes.

**Methods:**

We conducted a retrospective national cohort study using data from the Myocardial Ischaemia National Audit Project (MINAP), linked to hospital admission and mortality registries in England and Wales between 2005 and 2019. Frailty was assessed using the Secondary Care Administrative Records Frailty (SCARF) index and categorised as fit, mild, moderate, or severe. Multivariable Cox proportional hazards models were used with a primary outcome of all-cause mortality at 1-year.

**Findings:**

Of 931,133 patients with AMI, 317,967 (34.1%) were female. Frailty was more prevalent in females than in males (severe frailty: 53,065 [16.7%] vs. 64,018 [10.4%]). Males received more intensive therapeutic care across all frailty levels. After multivariable adjustment, the relationship between severe frailty and 1-year all-cause mortality was 26% greater in males than in females (relative hazard ratio [rHR]: 1.26, 95% CI 1.19–1.32, P-interaction <0.001). This corresponded to an adjusted absolute risk difference of 1.19% (95% CI 0.58%–1.79%)

**Interpretation:**

In this national AMI cohort, while frailty was more prevalent in females, its association with 1-year mortality was significantly greater in males. This sex-specific effect of frailty challenges current risk-assessment paradigms and underscores the need for sex-informed care pathways.

**Funding:**

10.13039/501100000272National Institute for Health and Care Research and British Heart Foundation Centre of Research Excellence, Leicester.

**Research in context**

**Evidence before this study**

Frailty is a well-established predictor of adverse outcomes after acute myocardial infarction (AMI), but evidence on sex-specific associations has been limited and inconsistent. We searched PubMed from inception up to 05/10/2025 using the terms “frailty”, “sex”, “acute myocardial infarction”, and “acute coronary syndrome”, identifying three key studies that formally tested this interaction. In a sub-study of the TRILOGY ACS trial of 4999 medically managed patients, a trend towards a greater prognostic impact of frailty on mortality was observed in men, though a formal interaction was not statistically significant. A Spanish single-centre cohort of 488 patients found that frailty was independently associated with long-term mortality in men but had a neutral effect in women. In contrast, a study of 535 octogenarians found that while both prefrailty and frailty predicted mortality in men, only severe frailty was prognostic in women, though with a very high hazard ratio. All studies noted a higher prevalence of frailty among women. Overall, the available data suggest that sex may modify the effect of frailty after AMI, but findings are based on small or selected populations with varying results.

**Added value of this study**

This population-based study of over 900,000 patients provides the largest and most definitive evidence base on the sex-specific effects of frailty in AMI. We found that while women had a higher prevalence of severe frailty, men within the same frailty strata presented with a higher burden of atherosclerosis-related comorbidities (e.g., diabetes) and more acute clinical instability (e.g., cardiac arrest). Despite receiving more intensive guideline-directed care, frailty was associated with a significantly greater relative increase in the risk of 1-year all-cause mortality and other adverse events in men compared to women. This “sex-frailty paradox” was robust, persisting across both STEMI and NSTEMI presentations.

**Implications of all the available evidence**

The combined evidence now clearly shows that while frailty is more prevalent in women with AMI, its prognostic impact on mortality is significantly greater in men. This disparity is not explained by differences in the intensity of care. This suggests that the biological meaning of a frailty diagnosis in the context of an AMI differs between the sexes and challenges a “one-size-fits-all” approach to risk assessment. Clinicians should be aware that frailty in men may signal a particularly high-risk cardiovascular phenotype. These findings underscore the need to integrate both frailty status and sex into clinical evaluation to facilitate more precise and equitable care pathways for all patients with AMI.

## Introduction

Acute myocardial infarction (AMI) remains a leading cause of global cardiovascular mortality and morbidity.^1^ The contemporary management of AMI is increasingly complicated by the growing prevalence of frailty within an ageing population with multiple long-term conditions (also known as multimorbidity).^2^ Frailty, a multidimensional clinical syndrome of diminished physiological reserves, is frequently observed in this patient group, affecting up to half of all individuals with AMI.^3^ Additionally, frailty is also a powerful, independent predictor of adverse outcomes including mortality, rehospitalisation and recurrent cardiovascular events in the general population.^4^, ^5^, ^6^

The intersection of frailty and sex in AMI presents a significant clinical and scientific challenge.^7^ It is well-established that females presenting with AMI have a higher burden of frailty compared to males.^8^ However, it remains uncertain whether the well-documented sex disparities in clinical outcomes are related to differing baseline frailty.^9^ An alternative hypothesis is that the effect of frailty itself is modified by sex. It is essential to distinguish between these possibilities particularly if frailty's impact is uniform, higher event rates in females may be explained by their greater frailty burden. Conversely, if frailty is a more potent risk marker in one sex, it would point towards fundamental biological differences and necessitate distinct, sex-specific approaches to risk assessment. To date, the intersection between frailty and sex, and its association with adverse outcomes following AMI has not been robustly investigated in a large, contemporary cohort. This study aims to examine sex differences in frailty and assess whether sex modifies the association between frailty and adverse outcomes in patients with AMI.

## Methods

### Study setting

This population-based study utilised linked electronic healthcare records from three national datasets in England and Wales. The Myocardial Ischaemia National Audit Project (MINAP) is a mandatory national registry enrolling patients with a diagnosis of type 1 AMI from all National Health Service (NHS) hospitals in England and Wales, capturing detailed demographic, comorbidity, and in-hospital treatment data.^10^ Using unique pseudonymised patient identifiers, MINAP records were linked to the Hospital Episode Statistics Admitted Patient Care (HES-APC) database, which contains diagnostic and procedural codes for all hospital admissions in England.^11^ All-cause mortality data were obtained via linkage to the Office for National Statistics (ONS), the official death registry, providing robust, long-term follow-up. This data linkage provides a comprehensive patient pathway from acute presentation to post-discharge outcomes.

Ethical approval was granted by the Health and Care Research Wales (HCRW) and the Health Research Authority (HRA Research Ethics Committee Reference 20/WA/0312). Further approval was also granted by the Confidentiality Advisory Group (CAG), an independent committee that provides advisory support on the use of confidential patient information for research.^12^^,^^13^ This study was reported in accordance with the Strengthening the Reporting of Observational Studies in Epidemiology (STROBE) guidelines.^14^

### Study population

We included all adult patients (≥18 years) admitted with a final diagnosis of AMI recorded in the MINAP registry between January 1, 2005, and December 31, 2019, with ONS mortality follow-up available until March 31, 2020. Patients were excluded if there were missing data for core administrative variables required for analysis, including age, sex, and admission date (Supplementary Figure S1).

### Frailty index

The Secondary Care Administrative Records Frailty (SCARF) index, which has been previously validated in HES-APC was used to measure frailty.^15^ The SCARF index measures cumulative deficit across 31 domains. It is a comprehensive measure of frailty and includes deficits found in both MINAP and HES-APC. Patients were grouped according to four levels of fitness based on the total frailty index value: Fit (FI = 0–0.05), Mild frailty (FI = 0.06–0.11), Moderate Frailty (FI = 0.12–0.18) and Severe frailty (FI ≥ 0.19). The SCARF index was used due to its strong predictive validity and comprehensive coverage of 365 ICD-10 codes, which is substantially broader than other administrative tools, such as the Hospital Frailty Risk Score (HFRS, 109 codes).^15^^,^^16^ While HFRS is widely applied, it has shown limitations in predicting outcomes in critically ill patients, a consideration relevant to our AMI cohort.^17^ In contrast, the SCARF index incorporates explicitly a wide range of cardiovascular comorbidities—such as hypertension, arrhythmias, diabetes, and heart failure—making it exceptionally well-suited for characterising frailty in this specific population. SCARF ICD-10 codes were extracted from the HES-APC admissions recorded at the time of each patient's inclusion in the MINAP registry. The Charlson Comorbidity Index (CCI) was calculated using similar approach to quantify baseline comorbidity burden.^18^ Details of the ICD-10 codes from HES-APC and the MINAP variables used to derive SCARF and CCI are provided in Supplementary Table S1.

### Quality of care

Quality of care during the index AMI admission was assessed using the European Society of Cardiology (ESC) Association for Acute Cardiovascular Care quality indicators (QIs) for AMI.^19^ Indicators included were: prescription of ACE inhibitors or ARBs, beta-blockers, dual antiplatelet therapy, and statins at discharge; door-to-balloon time of 1 h or less for ST-elevation myocardial infarction (STEMI); revascularization via PCI or CABG; invasive coronary angiography within 24 h for NSTEMI; and referral for cardiac rehabilitation.

### Outcome measures

The primary outcome was all-cause mortality at 1-year. Secondary outcomes were cardiovascular death, major adverse cardiovascular events (MACE), heart failure readmission, reinfarction, major bleeding and minor bleeding (Supplementary Table S1). Major adverse cardiovascular events were defined as a composite of all-cause mortality, reinfarction or rehospitalisation due to heart failure or ischaemic stroke.^20^

### Statistical analysis

Continuous variables are presented as medians and interquartile ranges (IQRs), while dichotomous and categorical variables are presented as total counts and percentages. Missing data were addressed using multiple imputation with chained equations, assuming missingness at random, generating ten imputed datasets with results combined using Rubin's rules. The imputation model specification is outlined in Supplementary Table S2.

Cox proportional hazard models were used to calculate adjusted hazard ratios (and 95% confidence intervals) with time to each outcome within 1 year of index AMI for both males and females, with ‘fit’ patients as the reference category. We aimed to adjust for the effect of cardiac rehabilitation on long-term (1-year) outcomes in frail patients, as it is a well-established therapy for frailty.^21^ Since cardiac rehabilitation is delivered out of hospital, it cannot influence in-hospital outcomes. To account for this, we employed a landmark analysis for 1-year outcomes, using time-to-event from discharge as the timescale in our Cox proportional hazards models, excluding in-hospital events. To assess the potential impact of selection bias introduced by this approach, we conducted a sensitivity analysis of 30-day outcomes, including in-hospital events occurring prior to discharge and excluding cardiac rehabilitation as a covariate. Given time to event was not available for in hospital events reported in MINAP, logistic regression was used to calculate an adjusted odds ratio (aOR) for events at 30-days.

All analyses were stratified by sex and additionally adjusted for age, ethnicity, smoking, ST-segment elevation on presentation, elevated myocardial enzymes, creatinine, Killip class, left ventricular (LV) function, hypercholesterolaemia, cardiac arrest, family history of coronary artery disease (CAD), in-patient revascularization (with PCI or CABG), in-hospital pharmacotherapy (including dual antiplatelet therapy, anticoagulation with fondaparinux, low molecular weight heparin or warfarin, glycoprotein IIb/IIIa, angiotensin converting enzyme or aldosterone receptor blockers, beta-blockers, mineralocorticoid antagonists and statins) and year of admission (modelled as a continuous variable). The main 30-day and 1-year analyses accounted for the hierarchical structure of the data, with patients clustered within operating centres. Population-averaged adjusted predicted probabilities were obtained using the margins command, with centre-level clustered standard errors to allow for arbitrary within-centre correlation. For time-to-event outcomes, Cox proportional hazards models incorporated a shared frailty term at the hospital level, which introduced a random effect to capture unobserved heterogeneity between centres. Proportional hazards assumptions were assessed using Schoenfeld residuals. To evaluate potential multicollinearity among covariates, the Variance Inflation Factor (VIF) was calculated for each variable. All VIFs were below the conventional threshold of 5, indicating that multicollinearity did not significantly affect the stability or precision of the model estimates.

To determine the relationship between frailty status and sex on outcomes, interaction terms between frailty and sex were incorporated to assess potential effect modification and are reported as relative hazard and odds ratios (with 95% confidence intervals [CIs]) for 1-year and 30-day outcomes respectively, using females as the reference group. P-interaction values were calculated to evaluate the statistical significance of the interaction terms. Sex-specific adjusted predicted probabilities and adjusted absolute risk differences (AARD, female minus male) were derived from the fitted models. Estimates are presented with 95% confidence intervals derived from the cluster-robust variance estimator for logistic models and shared frailty Cox models.

To assess any differential effects by AMI subtype, a pre-specified subgroup analysis was also performed for all outcomes stratified by sex and AMI subtype (ST-elevation myocardial infarction [STEMI] and Non-ST-elevation myocardial infarction [NSTEMI]). All statistical analyses were performed using Stata 18 (StataCorp).

### Role of the funding source

The funding sources had no role in the study design; the collection, analysis, or interpretation of data; the writing of the report; or the decision to submit for publication.

## Results

**Fig. 1:**
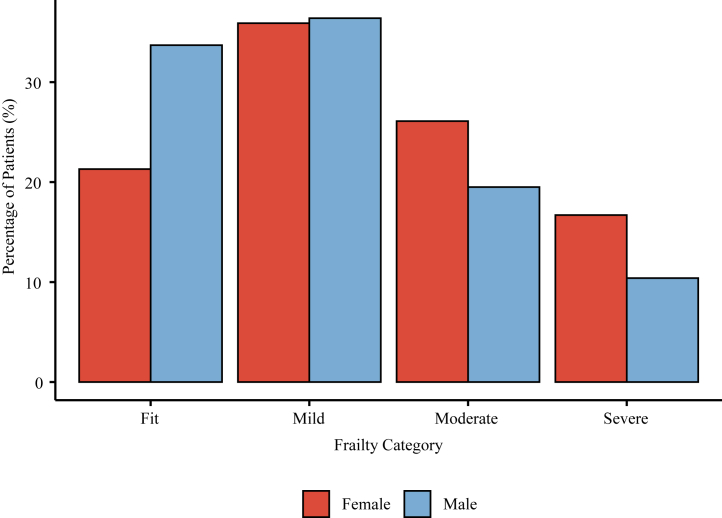
Comparison of levels of frailty between male and female patients with AMI.

A total of 931,133 patients with AMI were analysed, of whom 613,166 (65.9%) were male and 317,967 (34.1%) were female. The distribution of frailty differed by sex, with a higher proportion of females classified as severely frail compared to males (16.7% vs. 10.4%). In contrast, a larger proportion of males were categorised as fit (33.7% vs. 21.3%) (Fig. 1). Females were consistently older than males within each frailty stratum (median age for severely frail: 82 vs. 78 years). The patient cohort was predominantly white, although the proportion was slightly higher among females (fit females, 96.2% vs. fit males, 92.6%) (Table 1).

**Table 1:** Baseline characteristics of patients with AMI stratified by sex and frailty severity.

	Male				Female			
	Fit	Mild frailty	Moderate frailty	Severe frailty	Fit	Mild frailty	Moderate frailty	Severe frailty
Number of Patients (percentage)	206,433 (33.7%)	222,960 (36.4%)	119,755 (19.5%)	64,018 (10.4%)	67,603 (21.3%)	114,229 (35.9%)	83,070 (26.1%)	53,065 (16.7%)
Demographics and Co-morbidities								
Age, median (IQR)	59 (51–68)	67 (57–76)	74 (65–82)	78 (70–84)	67 (56–77)	75 (65–83)	79 (71–86)	82 (75–87)
Ethnicity								
White	83,476 (92.6%)	100,242 (91.0%)	60,465 (90.8%)	37,213 (90.2%)	27,075 (96.2%)	49,186 (94.0%)	40,218 (92.9%)	30,377 (91.8%)
Black	820 (0.9%)	1152 (1.0%)	707 (1.1%)	563 (1.4%)	218 (0.8%)	515 (1.0%)	499 (1.2%)	519 (1.6%)
Asian	5825 (6.5%)	8765 (8.0%)	5394 (8.1%)	3458 (8.4%)	838 (3.0%)	2649 (5.1%)	2565 (5.9%)	2189 (6.6%)
BMI, median (IQR)	27.0 (24.5–29.9)	27.4 (24.6–30.7)	27.4 (24.3–31.0)	27.3 (24.0–31.1)	26.1 (23.1–29.8)	26.5 (23.1–30.5)	26.5 (22.7–31.2)	26.2 (22.3–31.1)
Current smoker	80,302 (41.3%)	58,584 (28.2%)	22,598 (20.4%)	8932 (15.3%)	21,994 (35.1%)	23,344 (22.3%)	11,750 (15.6%)	5193 (11.0%)
Diabetes	7097 (3.4%)	51,477 (23.1%)	49,710 (41.5%)	36,496 (57.0%)	1988 (2.9%)	21,461 (18.8%)	28,264 (34.0%)	25,574 (48.2%)
Hypertension	45,375 (22.0%)	142,825 (64.1%)	91,512 (76.4%)	53,115 (83.0%)	18,284 (27.0%)	73,837 (64.6%)	63,392 (76.3%)	44,156 (83.2%)
Hypercholesterolaemia	40,765 (22.2%)	79,084 (39.3%)	46,227 (42.2%)	24,708 (41.7%)	12,968 (21.6%)	34,776 (33.7%)	27,090 (35.6%)	17,589 (35.9%)
Peripheral vascular disease	1728 (0.8%)	12,879 (5.8%)	18,191 (15.2%)	18,853 (29.4%)	464 (0.7%)	4436 (3.9%)	8063 (9.7%)	10,046 (18.9%)
Chronic kidney disease	643 (0.3%)	9323 (4.2%)	21,256 (17.7%)	28,247 (44.1%)	203 (0.3%)	4597 (4.0%)	12,406 (14.9%)	19,785 (37.3%)
History of respiratory disease	11,368 (5.5%)	43,523 (19.5%)	42,776 (35.7%)	33,925 (53.0%)	5314 (7.9%)	27,495 (24.1%)	31,244 (37.6%)	27,858 (52.5%)
Heart failure	8742 (4.2%)	39,970 (17.9%)	47,691 (39.8%)	41,763 (65.2%)	2130 (3.2%)	17,977 (15.7%)	30,761 (37.0%)	32,421 (61.1%)
Stroke/TIA	1323 (0.6%)	12,468 (5.6%)	19,009 (15.9%)	18,885 (29.5%)	614 (0.9%)	7405 (6.5%)	13,177 (15.9%)	15,008 (28.3%)
Cognitive and mental health problems	1332 (0.6%)	6739 (3.0%)	8691 (7.3%)	10,510 (16.4%)	940 (1.4%)	6472 (5.7%)	9825 (11.8%)	12,014 (22.6%)
Family history of CAD	62,774 (39.1%)	55,197 (32.9%)	22,033 (25.2%)	8651 (18.9%)	19,359 (37.8%)	25,234 (30.2%)	13,573 (22.9%)	6416 (17.3%)
History of ischaemic heart disease	16,321 (7.9%)	91,880 (41.2%)	72,887 (60.9%)	45,472 (71.0%)	4172 (6.2%)	36,517 (32.0%)	40,773 (49.1%)	31,583 (59.5%)
Charlson comorbidity index								
CCI = 0–1	197,044 (95.5%)	141,757 (63.6%)	27,990 (23.4%)	4249 (6.6%)	65,357 (96.7%)	81,835 (71.6%)	27,778 (33.4%)	6615 (12.5%)
CCI = 2–3	8377 (4.1%)	74,877 (33.6%)	63,740 (53.2%)	20,137 (31.5%)	1862 (2.8%)	30,349 (26.6%)	43,020 (51.8%)	21,037 (39.6%)
CCI > 3	1012 (0.5%)	6326 (2.8%)	28,025 (23.4%)	39,632 (61.9%)	384 (0.6%)	2045 (1.8%)	12,272 (14.8%)	25,413 (47.9%)
Clinical characteristics								
Left ventricular ejection fraction (echocardiogram)								
Good (≥50%)	57,008 (65.9%)	55,213 (55.7%)	25,124 (43.9%)	11,438 (34.0%)	17,767 (68.2%)	27,698 (60.8%)	18,398 (51.9%)	11,137 (44.6%)
Moderate (30–49%)	25,383 (29.3%)	33,972 (34.3%)	21,762 (38.0%)	13,498 (40.1%)	7069 (27.1%)	13,978 (30.7%)	12,210 (34.4%)	9206 (36.8%)
Poor (<30%)	4109 (4.8%)	9959 (10.0%)	10,393 (18.1%)	8728 (25.9%)	1216 (4.7%)	3878 (8.5%)	4842 (13.7%)	4652 (18.6%)
Systolic blood pressure, median (IQR)	139 (122–156)	137 (120–156)	135 (117–154)	131 (113–152)	140 (121–158)	140 (120–160)	139 (119–160)	136 (116–158)
Heart rate, median (IQR)	73 (63–85)	76 (64–90)	80 (67–95)	82 (69–98)	77 (66–89)	80 (68–94)	83 (70–99)	85 (71–100)
Creatinine umol/L, median (IQR)	86 (75–98)	90 (77–108)	101 (83–131)	124 (94–173)	70 (61–83)	76 (64–94)	86 (69–112)	100 (76–138)
Killip class								
Killip class I	73,644 (79.5%)	76,082 (59.5%)	33,828 (40.0%)	13,247 (25.4%)	21,416 (68.7%)	31,886 (47.2%)	19,414 (32.9%)	9947 (23.2%)
Killip class II	16,919 (18.3%)	44,093 (34.5%)	42,324 (50.0%)	30,256 (58.1%)	8779 (28.2%)	31,200 (46.2%)	33,471 (56.7%)	26,176 (60.9%)
Killip class III	688 (0.7%)	2533 (2.0%)	3787 (4.5%)	4798 (9.2%)	352 (1.1%)	1632 (2.4%)	2912 (4.9%)	3935 (9.2%)
Killip class IV	1405 (1.5%)	5267 (4.1%)	4676 (5.5%)	3763 (7.2%)	610 (2.0%)	2865 (4.2%)	3261 (5.5%)	2895 (6.7%)
STEMI	103,075 (49.9%)	85,822 (38.5%)	33,757 (28.2%)	13,215 (20.6%)	28,398 (42.0%)	37,278 (32.6%)	21,004 (25.3%)	10,921 (20.6%)
NSTEMI	103,358 (50.1%)	137,138 (61.5%)	85,998 (71.8%)	50,803 (79.4%)	39,205 (58.0%)	76,951 (67.4%)	62,066 (74.7%)	42,144 (79.4%)
Cardiac arrest	9834 (4.9%)	14,274 (6.6%)	9729 (8.4%)	6159 (9.9%)	3221 (4.9%)	6156 (5.6%)	5189 (6.5%)	3730 (7.2%)

Patients with missing data excluded from calculation of percentages, medians and IQRs.

### Baseline characteristics

The prevalence of most comorbidities, such as hypertension and heart failure, increased with the severity of frailty for both sexes. However, within severe frailty levels, males had a higher prevalence of diabetes (57.0% vs. 48.2%) and a greater aggregate comorbidity burden (Charlson Comorbidity Index >3: 61.9% vs. 47.9%). Acutely, severely frail males presented more frequently with cardiac arrest (9.9% vs. 7.2%), while severely frail females more often had preserved left ventricular function (LVEF ≥50%: 44.6% vs. 34.0%). While the mean SCARF index increased with age for both sexes, younger females typically exhibited higher frailty levels than younger males, that attenuated with advancing age (Supplementary Figure S2). Between 2005 and 2019, the overall AMI population became frailer, but the sex-gap in frailty prevalence persisted (Supplementary Figure S3).

### In-hospital care

The use of guideline-recommended medications and interventions declined with increasing frailty in both males and females, as shown in Table 2 and Supplementary Figure S4. Within each frailty category, however, males received key therapies more frequently compared to females. Among severely frail patients, males were more likely than females to undergo invasive coronary angiography (49.6% vs. 38.5%) and percutaneous coronary intervention (PCI) (36.9% vs. 29.7%). Consequently, composite quality of care scores were also higher for severely frail males than for females in the same category (mean OBQI score: 81.7 vs. 77.7). The median length of hospital stay increased with frailty for both sexes and was slightly longer for females in the frailer categories (severe frailty: 9 days in females compared to 8 days in males).

**Table 2:** Summary of in-hospital care including ESC AMI quality of care indicators stratified by sex and frailty status.

	Male				Female			
	Fit	Mild frailty	Moderate frailty	Severe frailty	Fit	Mild frailty	Moderate frailty	Severe frailty
Length of hospital stay (days), median (IQR)	4 (2–6)	4 (2–7)	5 (3–10)	8 (4–15)	4 (2–7)	5 (3–8)	6 (4–11)	9 (5–16)
Medications prescribed								
Dual antiplatelets	167,956 (94.2%)	173,907 (89.7%)	87,797 (83.5%)	43,714 (77.3%)	51,948 (91.0%)	83,097 (85.6%)	56,841 (79.3%)	34,083 (73.3%)
Fondaparinux or LMWH	118,059 (69.3%)	135,253 (72.6%)	75,449 (74.0%)	40,413 (73.5%)	40,981 (73.0%)	72,632 (75.5%)	54,232 (76.2%)	34,577 (75.2%)
Unfractionated heparin	56,048 (33.9%)	49,684 (27.5%)	21,559 (21.7%)	9639 (17.9%)	14,962 (27.6%)	19,644 (21.1%)	11,312 (16.3%)	6262 (13.9%)
Warfarin	2917 (1.8%)	7947 (4.4%)	8978 (9.0%)	6612 (12.2%)	1049 (1.9%)	4142 (4.4%)	5377 (7.7%)	4517 (10.0%)
Glycoprotein IIb/IIIa inhibitors	21,452 (12.7%)	17,384 (9.4%)	6521 (6.4%)	2316 (4.2%)	5067 (9.1%)	6076 (6.4%)	3066 (4.4%)	1315 (2.9%)
ACE inhibitor or ARB	154,206 (89.5%)	166,671 (86.8%)	84,798 (81.4%)	39,972 (73.1%)	46,142 (82.8%)	78,862 (81.0%)	54,924 (76.6%)	31,545 (70.0%)
Beta-blocker	165,790 (90.3%)	170,099 (85.1%)	85,926 (80.2%)	44,756 (78.9%)	51,736 (86.7%)	81,351 (80.3%)	56,471 (76.5%)	35,679 (76.2%)
Statin	170,242 (96.9%)	186,875 (95.0%)	98,956 (92.4%)	51,240 (90.1%)	53,334 (94.0%)	89,939 (90.9%)	63,752 (87.8%)	39,322 (85.1%)
Mineralocorticoid receptor antagonist	6600 (6.3%)	11,935 (10.0%)	10,554 (15.3%)	7774 (19.8%)	1776 (5.6%)	4837 (8.5%)	5617 (12.4%)	5198 (16.1%)
Interventional management								
Invasive coronary angiogram	125,103 (72.4%)	120,311 (66.5%)	52,107 (57.6%)	21,121 (49.6%)	32,072 (59.5%)	45,534 (51.6%)	26,045 (43.7%)	12,789 (38.5%)
PCI	110,420 (63.9%)	100,447 (55.6%)	40,683 (45.0%)	15,730 (36.9%)	27,878 (51.7%)	37,962 (43.0%)	20,699 (34.7%)	9864 (29.7%)
CABG surgery	4324 (2.5%)	6144 (3.4%)	3222 (3.6%)	1477 (3.5%)	738 (1.4%)	1526 (1.8%)	1096 (1.9%)	552 (1.7%)
Other ESC AMI quality of care indicators								
Time to angiography <24 h (NSTEMI)	15,585 (37.6%)	16,099 (35.4%)	7086 (31.7%)	2848 (28.0%)	3855 (34.5%)	5822 (32.6%)	3072 (28.0%)	1395 (24.4%)
Door to balloon time <1 h (STEMI)	64,968 (77.2%)	48,769 (73.5%)	16,313 (70.0%)	4734 (63.0%)	16,158 (74.7%)	18,449 (71.6%)	8443 (68.7%)	3248 (62.4%)
Admitted to cardiology ward	151,297 (75.2%)	149,705 (69.2%)	71,278 (61.8%)	33,302 (54.7%)	44,780 (67.9%)	67,230 (60.3%)	43,042 (53.2%)	23,845 (46.4%)
Rehabilitation referral made	164,955 (90.7%)	162,755 (85.7%)	79,168 (81.1%)	36,411 (75.0%)	50,251 (86.5%)	76,136 (80.2%)	49,430 (74.7%)	27,089 (68.0%)
OBQI score, mean (SD)	93.6 (14.6)	90.5 (16.5)	86.5 (18.8)	81.7 (20.9)	90.4 (17.7)	86.7 (19.3)	82.5 (21.1)	77.7 (23.0)

Patients with missing data excluded from calculation of percentages, medians and IQRs.

### Clinical outcomes

In unadjusted analyses (Supplementary Figure S5 and Supplementary Table S3), males who were fit had a lower absolute risk for the primary outcome of 1-year all-cause mortality compared to fit females (Absolute Risk [AR]: 2.7% vs. 4.6%), with an absolute risk difference (ARD) of −1.90% (95% CI −2.07% to −1.72%). This risk difference between sexes diminished as frailty increased, with the ARD narrowing to −1.31% (95% CI −1.88% to −0.74%) in the severely frail group. A similar pattern was observed for the key secondary outcome of 1-year MACE, where the initial lower risk in fit males (ARD: −2.24%, 95% CI −2.52% to −1.97%) was reduced and ultimately reversed among severely frail patients, where males had a higher absolute risk than females (AR: 45.1% vs. 44.2%; ARD: +0.95%, 95% CI 0.38%–1.52%).

### Adjusted outcomes

**Fig. 2:**
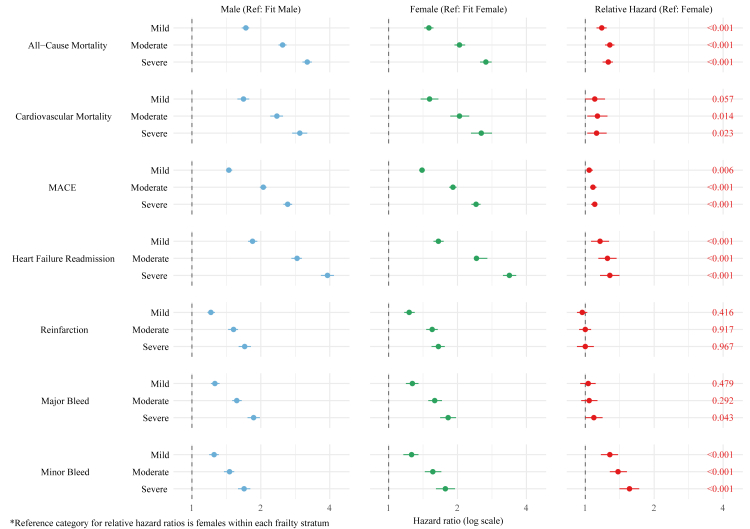
Adjusted 1-year outcomes in patients with AMI stratified by sex and frailty severity.

**Fig. 3:**
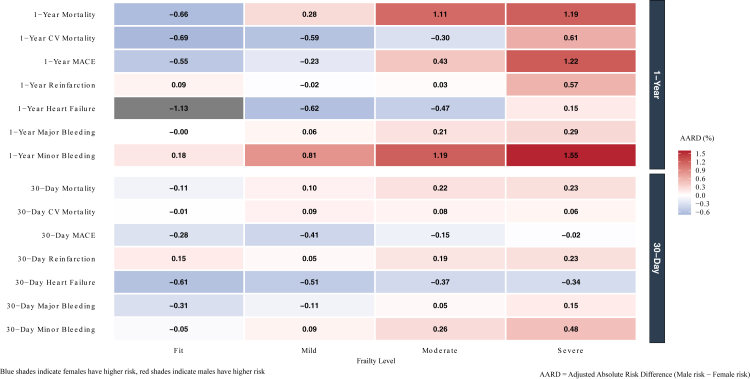
Visual illustration of adjusted absolute risk differences between males and females stratified frailty severity.

After multivariable adjustment, greater frailty at 1-year was associated with higher risks of all outcomes in both sexes; however, the relative strength of this association was generally greater in males. As summarised in Supplementary Table S4 and illustrated in Fig. 2, the adjusted hazard ratio (aHR) in males increased from 1.72 (95% CI 1.65–1.78) for mild frailty to 3.19 (95% CI 3.04–3.34) for severe frailty. In females, the aHR increased from 1.50 (95% CI 1.43–1.57) to 2.66 (95% CI 2.51–2.82) across the same frailty spectrum. Formal interaction testing confirmed that the association with severe frailty and 1-year mortality was 26% greater in males than in females (relative hazard ratio [rHR]: 1.26, 95% CI 1.19–1.32; P-interaction <0.001). As shown in Fig. 3 and Supplementary Table S5, analysis of model-based predicted probabilities demonstrated that the AARD for fit patients was −0.66% (95% CI: −1.04% to −0.27%), indicating a higher predicted probability of 1-year mortality among fit females compared with males. With increasing levels of frailty this pattern reversed, and in severely frail patients the AARD was 1.19% (95% CI: 0.58%–1.79%), indicating a significantly higher predicted probability of death in males. The heightened relationship between males with frailty and adverse outcomes also extended to 1-year MACE and heart failure readmission.

**Fig. 4:**
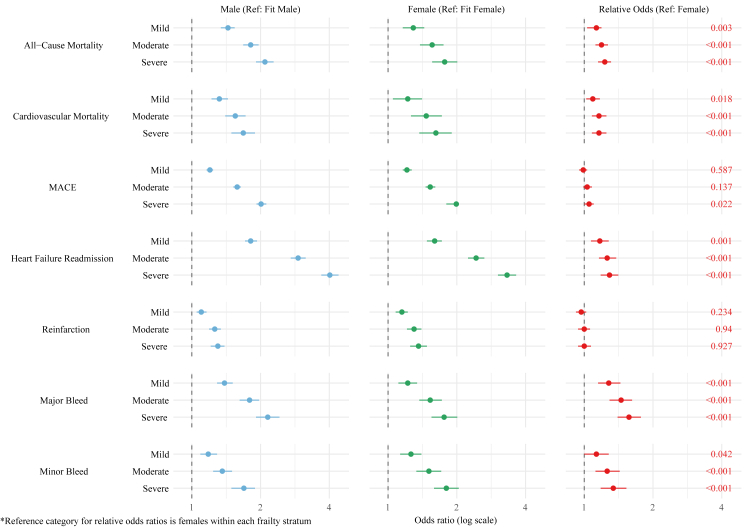
Adjusted 30-day outcomes in patients with AMI stratified by sex and frailty severity.

A similar sex interaction was observed for 30-day outcomes, shown in Fig. 4 and Supplementary Table S6. Increasing frailty was associated with a stepwise increase in the odds of adverse events for both males and females when compared to their fit counterparts. For 30-day all-cause mortality, the adjusted odds ratio (aOR) in males rose from 1.44 (95% CI 1.34–1.54) for mild frailty to 2.09 (95% CI 1.91–2.28) for severe frailty. Similarly, for females, the aOR increased from 1.29 (95% CI 1.16–1.44) to 1.77 (95% CI 1.56–2.01). A significant sex interaction was revealed, showing that the relative odds of all-cause mortality for severe frailty were 23% higher in males compared to females (relative odds ratio [rOR]: 1.23, 95% CI 1.15–1.31; P-interaction <0.001). Similar findings were observed for 30-day cardiovascular death and heart failure readmission. Analysis of predicted probabilities (Supplementary Table S5 and Fig. 3) showed that fit patients had an AARD of −0.11% (95% CI −0.27% to 0.04%), indicating a slightly higher 30-day mortality risk in females, whereas in severely frail patients the AARD was 0.23% (95% CI 0.01%–0.45%), indicating a marginally higher risk in males.

### Outcomes by AMI subtypes

At 1-year, the relationship between frailty and adverse outcomes amongst males remained consistent across both STEMI and NSTEMI cohorts for all-cause mortality (Supplementary Table S7). In STEMI patients with severe frailty, the aHR was 3.26 (95% CI 3.05–3.50) for males and 2.67 (95% CI 2.46–2.90) for females (rHR: 1.32, 95% CI 1.21–1.45). Similar observations were recorded in the NSTEMI cohort (rHR for severe frailty: 1.32, 95% CI 1.21–1.45). For 1-year MACE and heart failure readmission, the link between frailty appeared stronger in males regardless of AMI type.

At 30 days, the stronger relationship between frailty in males and all-cause mortality, was more pronounced in the STEMI cohort (rOR for severe frailty: 1.28, 95% CI 1.15–1.41) compared to the NSTEMI cohort (rOR for severe frailty: 1.13, 95% CI 1.02–1.24) (Supplementary Table S8). A key difference emerged for short-term cardiovascular death, where the relative increase in risk within frail males was significant only in the STEMI cohort (rOR for severe frailty: 1.23, 95% CI 1.10–1.37).

## Discussion

In this large, contemporary, fifteen-year national cohort of over 900,000 patients with AMI, our study provides several novel insights into the interplay between frailty and sex. Our first principal contribution is the detailed characterisation of the population, where we found that while females were chronologically older with a higher prevalence of severe frailty, males within the same frailty strata carried a greater burden of atherosclerosis-related comorbidities and presented more acutely. Second, despite this higher-risk clinical profile and receiving more intensive guideline-directed care, males exhibited a profoundly greater relative increase in risk of mortality and major adverse cardiovascular events with increasing frailty compared to females within the same frailty strata. Third, this “sex-frailty paradox”, whereby frailty is more prevalent in females but is linked to more severe outcomes in males, was consistent across both STEMI and NSTEMI presentations and persisted at 1-year.

Our findings that frailty is strongly associated with adverse outcomes after AMI align with an established body of literature.^2^^,^^3^ Seminal studies such as the LONGEVO-SCA registry and the MOSCA-FRAIL trial were critical in establishing the high prevalence and prognostic importance of frailty in elderly patients with NSTEMI.^22^^,^^23^ These studies have appropriately shifted the research focus towards treatment decisions in this high-risk group, such as whether invasive strategies benefit frail older adults. However, this prior work has often grouped patients by age, leaving the crucial interaction between frailty and sex largely unexplored. Our study builds directly on this foundation by demonstrating that the prognostic impact of frailty is not uniform, but is significantly modified by sex, a finding that has important implications for future trial design and for translating frailty assessment into clinical practice for all adults.

Our work in this high-acuity clinical population provides a distinct illustration of the “male-female health-survival paradox”.^24^ A large meta-analysis of general population studies confirmed this pattern, and importantly, this paradox has also been suggested in smaller, specific cardiovascular cohorts, including outpatients with heart failure.^25^^,^^26^ Our study is the first to confirm and quantify this paradox on a national scale in the specific context of an AMI.

The observed association between frailty and adverse outcomes in males may be rooted in fundamental differences in how the frailty syndrome manifests in this population.^7^ It is plausible that frailty in males with AMI is a surrogate for a more advanced and biologically unstable atherosclerotic phenotype.^27^ Our data support this, as frail males had a higher prevalence of diabetes and a greater aggregate comorbidity burden. Their more frequent presentation with cardiac arrest and cardiogenic shock suggests a more advanced and vulnerable cardiac state. This aligns with broader population data showing that while males have fewer chronic conditions overall, the ones they do have are more frequently life-threatening and directly linked to cardiovascular health, such as ischaemic heart disease and peripheral artery disease.^25^ The profound impact of frailty on males’ outcomes, even when they receive more intensive therapeutic care, suggests their vulnerability and diminished physiological reserves to withstand a major cardiovascular insult in the form of AMI.^28^ Conversely, frailty in females may represent a more global, multi-system decline not exclusively linked to the severity of their coronary disease.^29^ This suggests that frailty in females is a marker of accumulated disability across multiple systems rather than being driven primarily by advanced coronary disease. Their relative resilience to the index AMI, despite a higher frailty burden, may be explained by inherent sex differences in biology.^30^ Females are known to have different lifelong inflammatory and immune responses, and men experience immunosenescence (the aging of the immune system) at a faster rate.^31^ This may provide females with a greater capacity to withstand the acute inflammatory storm of a myocardial infarction.^32^

Our results show that the prevalence of severe frailty was substantially higher in females than in males, a gap that persisted over the fifteen-year study period despite a rise in frailty in the overall AMI population. This finding aligns with general population studies where females consistently have higher frailty scores.^25^ However, it raises the question of whether standard frailty tools, including administrative data-based indices like SCARF, systematically overestimate the severity of frailty in females.^33^ These tools often include variables influenced by sex-specific differences in body composition, muscle mass, and self-reported health deficits, which may not carry the same prognostic weight in females as in males.^34^ This could explain why frailty appears more common but less prognostically influential in females, suggesting that the same frailty score may represent a different level of physiological vulnerability in males and females.

Our findings have immediate and significant clinical and healthy policy implications. The current approach to risk stratification in AMI, which is heavily reliant on chronological age, is insufficient and must evolve to include routine frailty assessment for all patients.^35^ Frail males represent a particularly high risk group for whom standard post-AMI care pathways are currently inadequate. Their care should be enhanced beyond guideline directed medical therapy (GDMT) to include holistic, geriatric-led interventions, intensive cardio-metabolic management and prioritised cardiac rehabilitation.^36^ For frail females, the policy implications differ. Their high absolute risk combined with lower rates of GDMT use, indicates that they remain an undertreated population. Here, the primary policy priority is to close this persistent care gap and ensure equitable delivery of established, life-saving therapies.^9^

Our study also provides several critical avenues for future research. First, mechanistic studies are urgently needed to explore the biological underpinnings of the sex-frailty paradox, including sex differences in inflammation, immunosenescence, and skeletal muscle metabolism.^31^ Second, our findings indicate discrepancies in how current frailty assessment approaches capture risk in males versus females. The development and validation of new sex-specific frailty assessment tools should therefore be a priority for future research, as such tools may be better suited to identifying high-risk individuals and guiding targeted interventions for the most vulnerable patients. Finally, multi-component interventions are essential. Recent trial data have shown substantial benefits of comprehensive rehabilitation programs, incorporating both exercise and nutritional support, in older adults with impaired physical function following AMI.^37^ Our findings suggest that future trials of such multi-domain interventions should be stratified by both sex and frailty. This approach is necessary to account for the clear sex-specific differences we observed and to determine if the particularly poor trajectory of these subgroups can be modified.

This study's primary strength lies in its use of a large, contemporary, and nationally representative cohort, with robust linkage across clinical, hospitalisation, and mortality datasets. The use of the validated SCARF index enabled objective and standardised frailty assessment across the entire population using routinely collected data.^15^ The SCARF index also comprehensively assesses frailty in a broader sense than other frailty scores. However, our study has potential limitations. First, the potential for unmeasured confounding variables, such as socioeconomic status or functional capacity, remains. Second, the SCARF index relies on the accuracy and consistency of hospital coding practices, which may lead to some misclassification of frailty. Third, SCARF might not be well-suited for the clinical frontline as it will depend on integrated systems to readily automate and calculate patients' indices based on already coded data, to guide clinical decisions. This is not readily available in many health systems and simpler models of frailty assessment might be easily adopted by clinicians making decisions regarding inpatient care. Fourth, these findings are based on a UK-based registry and may require validation in healthcare systems with different data infrastructures and care delivery models. Although absolute event rates may vary across settings, we postulate that the underlying biological interaction between frailty and sex is likely to remain consistent. Finally, the study period ended just prior to the onset of the COVID-19 pandemic. While this limits generalisability to current practice in the pandemic and post-pandemic era, it was a deliberate methodological choice. This 15-year pre-pandemic cohort provides a stable baseline for examining the underlying sex-frailty interaction, free from the significant confounding and systemic shocks to care delivery introduced by the pandemic, which would require a separate, dedicated analysis.

### Conclusion

In this large, national cohort of patients with acute myocardial infarction, frailty was more strongly associated with 1-year mortality in males than in females, despite being more prevalent in females. Males experienced worse outcomes despite receiving more intensive guideline-directed care, suggesting this disparity may be driven by underlying biological differences rather than treatment gaps alone. Our findings suggest considering both frailty and sex in routine clinical evaluation may be useful in developing more precise and equitable care pathways for all patients with AMI.

## Contributors

Conceptualisation, MR, HM, and CHM; methodology, MR and HM; formal analysis, MR and HM; data curation, MR and HM; writing–original draft preparation, MR, HM, and CHM; writing–review and editing, MR, HM, CHM, CL, KK, AW, IBS, GPM, AAD, SB, MAK, and MAM; supervision, MR. HM and CHM contributed equally to the study.

## Data sharing statement

The authors do not have authorisation to share the data used for this study. Data can be accessed through contacting the National Institute for Cardiovascular Outcomes Research (NICOR) and National Health Service (NHS) Digital.

## Declaration of interests

The authors declare no conflicts of interest related to the present study. KK reports consultancy relationships with Amgen, AstraZeneca, Bristol Myers Squibb, Boehringer Ingelheim, Lilly, Novo Nordisk, Sanofi, Servier, Pfizer, Roche, Daiichi-Sankyo, Embecta and Nestle Health Science; AAD reports receiving funding from the National Heart, Lung, and Bone Institute; and GPM reports receiving funding from the British Heart Foundation (BHF) and National Institute for Health and Care Research (NIHR) in addition to a leadership role in the British Society of Cardiovascular Magnetic Resonance Research Group, all outside the submitted work.
