# Supplementary material for: Sex–specific associations between frailty and long-term outcomes in patients with acute myocardial infarction: a national population-based study
Source: Lancet Reg Health Eur. 2026 Feb 12;64:101612. doi: 10.1016/j.lanepe.2026.101612 (PMC12925122; doi:10.1016/j.lanepe.2026.101612)
Supplement: Supplementary Figures and Tables [file mmc1.pdf]

Supplementary Data Table of Contents

Supplementary Figure 1 – Page 2

Supplementary Table 1 – Page 3

Supplementary Table 2 – Page 5

Supplementary Figure 2 – Page 7

Supplementary Figure 3 – Page 8

Supplementary Figure 4 – Page 9

Supplementary Figure 5 – Page 10

Supplementary Table 3 – Page 11

Supplementary Table 4 – Page 12

Supplementary Table 5 – Page 13

Supplementary Table 6 – Page 15

Supplementary Table 7 – Page 16

Supplementary Table 8 – Page 18

Supplementary Figure 1: STROBE diagram outlining process of cohort selection from linked MINAP Database

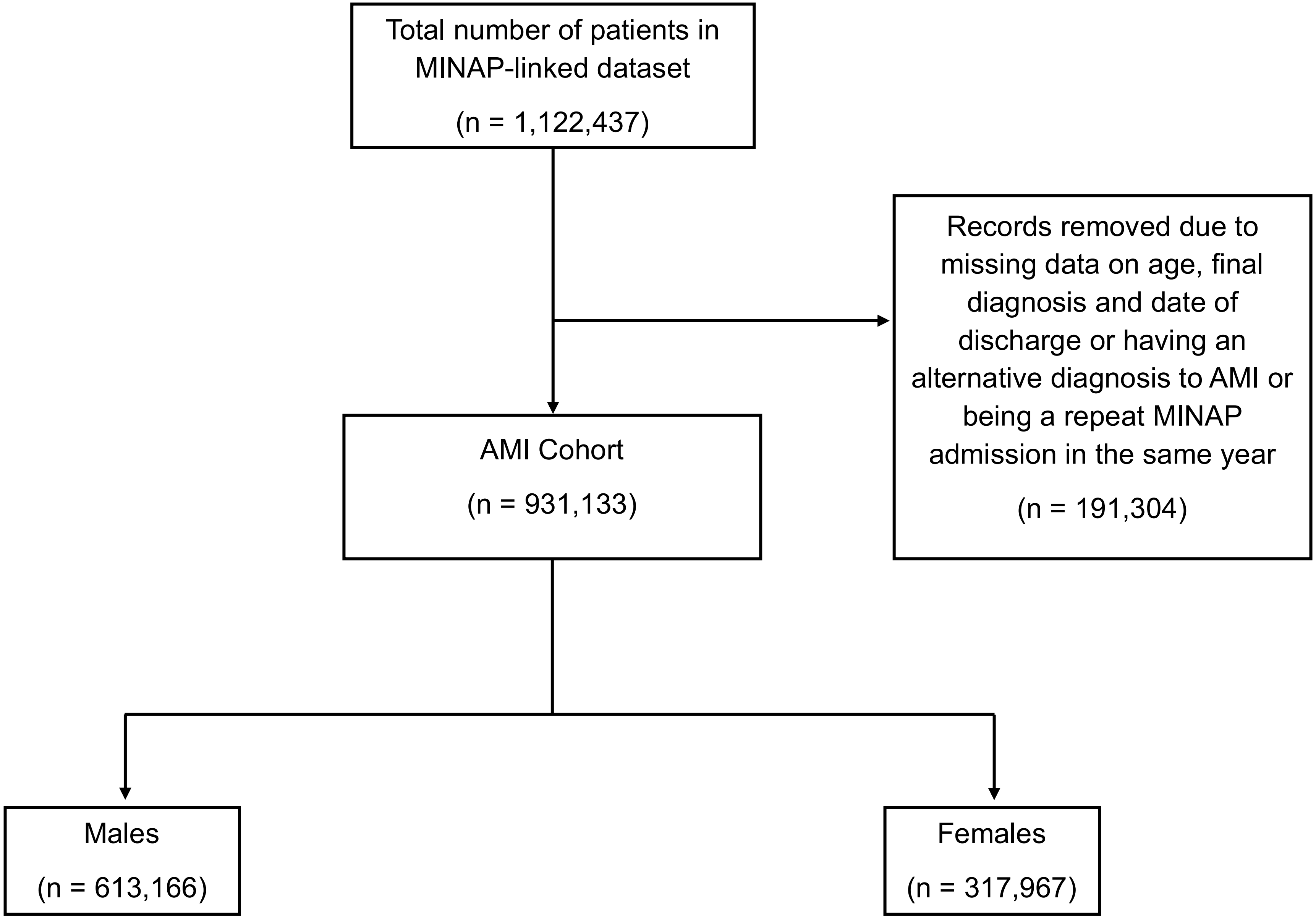

Supplementary Table 1: Summary of ICD-10 codes and MINAP variables used to extract data on SCARF Index, Charlson's Co-morbidity Index and outcomes

**Secondary Care Administrative Records Frailty Index**	
Activity Limitation	R26 S78 S88 Y83 G11 G81 G82 G83 M62 T136 Z993
Neurodegenerative Disorders	G20 G21 G22 G23 G25 G26 G32 G35 R25 G122
Requirement for Care	R40 Z50 Z74 Z755 Z998 Z999
Social Vulnerability	F10 Y06 Z59 Z60 Z63 Z73 R460 R468
Hearing Impairment	H90 H91 H833 Z453 Z461 Z974
Visual Impairment	H25 H28 H35 H40 H43 H53 H54
Falls	S00 S01 W00 W01 W04 W05 W06 W07 W08 W10 W18 W19 R296
Skin Ulcer	I83 I98 L03 L08 L89 L97 L984
Incontinence	N31 R15 R32 N393 N394 T835 Z466
Nutritional Problems	E41 E43 E44 E46 E53 E55 E66 E83 E87 R53 R63 R64 X53 R628
Cognitive and Mental Health Problems	F00 F01 F02 F03 F04 F05 G30 G31 R41 R54 F2 F3 F41 R44 R45 F067
Anaemia	D50 D51 D52 D53 D63 D64
Arthritis	M05 M06 M07 M09 M10 M11 M12 M13 M15 M16 M17 M18 M19 M32 M34 M35 M36 M315
Cardiac Arrhythmias	I44 I48 I49 Z450 Z950
Cerebrovascular Disease	G45 G46 I60 I61 I62 I63 I64 I65 I66 I67 I68 I69<br>*MINAP variable: previous cerebrovascular disease*
Chronic Kidney Disease (CKD)	I12 I13 N01 N03 N05 N07 N08 N18 N19 N25 Z49 I770 Z940 Z992<br>*MINAP variable: chronic kidney disease*
Diabetes	E109 E119 E129 E139 E149<br>*MINAP variable: diabetes*
Diabetic Complications	E100 E101 E102 E103 E104 E105 E106 E107 E108 E110 E111 E112 E113 E114 E115 E116 E117 E118 E120 E121 E122 E123 E124 E125 E126 E127 E128 E130 E131 E132 E133 E134 E135 E136 E137 E138 E140 E141 E142 E143 E144 E145 E146 E147 E148 G590 G632 H360 M142 M146 N083
Heart Failure	I11 I13 I27 I42 I43 I50 I51 I260<br>*MINAP variable: history of heart failure*
Heart Valve Disease	I05 I06 I07 I08 I34 I35 I37 I390 I391 I392 I393 I394 Z952 Z953 Z954
Hypertension	I10 I11 I12 I13 H350<br>*MINAP variable: hypertension*
Hypotension	I95 R55 R42 E86<br>*MINAP variables: systolic blood pressure or Killip class IV*
Ischaemic Heart Disease (IHD)	I252<br>*MINAP variables: previous MI, previous PCI, previous CABG, previous angina*
Foot Problems	L60 S90 S91 S92 S93 S94 S96 S97 S99 Q66 B353 G575 G576 M201 M202 M203 M204 M205 M206 M213 M214 M215 M216 M722 M766 M773 M775
Fragility Fracture	S22 S32 S33 S42 S43 S62 S72 S73 M484
Osteoporosis	M80 M81 M82
Peptic Ulcer Disease	K21 K25 K26 K27 K29 R12
Peripheral Vascular Disease	I65 I70 I71 I72 I73 R02 I771 K551 K558 K559 Z958 Z959<br>*MINAP variable: history of peripheral vascular disease*
Respiratory Disease	J45 J46 J40 J41 J42 J43 J44 J47 J60 J61 J62 J63 J64 J65 J70 J13 J14 J15 J16 J18 J22 J90 R06 J684 J961 J980<br>*MINAP variable: history of asthma or COPD*
Thyroid Disease	E03 E04 E05 E06 E079
Urinary System Disease	N30 N34 R31 R33 N390 N398 N399 T835
**Charlson Co-morbidity Index**	
Myocardial Infarction	I254<br>*MINAP variable: previous MI*
Congestive Heart Failure	I50 I43 I09 I099 I110 I130 I132 I255<br>*MINAP variable: history of heart failure*

Peripheral Vascular Disease	I70 I71 I73 I77 I79 K55 Z95<br>*MINAP variable: history of peripheral vascular disease*
Cerebrovascular Disease	G45 G46 I60 I61 I62 I63 I64 I65 I66 I67 I68 I69 H34<br>*MINAP Variable: history of cerebrovascular disease*
Dementia	F00 F01 F02 F03 G30 F05 G31
Chronic Pulmonary Disease	I27 J40 J41 J42 J43 J44 J45 J46 J47 J60 J61 J62 J63 J64 J65 J66 J67 J68 J70<br>*MINAP variable: history of asthma or COPD*
Connective Tissue Disease	M05 M06 M315 M32 M33 M34 M351 M353 M360
Peptic Ulcer Disease	K25 K26 K27 K28
Hemiplegia/Paraplegia	G81 G82 G83
Diabetes without Complications	E10 E11 E12 E13 E14 E100 E101 E106 E108 E109 E110 E111 E116 E118 E119 E120 E121 E126 E128 E129 E130 E131 E136 E138 E139 E140 E141 E146 E148 E149<br>*MINAP variable: diabetes*
Diabetes with Complications	E102 E103 E104 E105 E107 E112 E115 E117 E122 E123 E124 E125 E127 E132 E133 E134 E135 E137 E142 E143 E144 E145 E147
Mild Liver Disease	B18 K70 K73 K74
Moderate/Severe Liver Disease	I850 I859 I864 I982 K704 K711 K721 K729 K765 K766 K767
Renal Disease	I12 I13 N03 N05 N18 N19 N25 Z49 Z94 Z99<br>*MINAP variable: history of chronic kidney disease*
Any Malignancy	C00 C01 C02 C03 C04 C05 C06 C07 C08 C09 C10 C11 C12 C13 C14 C15 C16 C17 C18 C19 C20 C21 C22 C23 C24 C25 C26 C30 C31 C32 C33 C34 C37 C38 C39 C40 C41 C43 C45 C46 C47 C48 C49 C50 C51 C52 C53 C54 C55 C56 C57 C58 C60 C61 C62 C63 C64 C65 C66 C67 C68 C69 C70 C71 C72 C73 C74 C75 C76 C81 C82 C83 C84 C85 C88 C90 C91 C92 C93 C94 C95 C96 C97
Metastatic Solid Tumor	C77 C78 C79 C80
HIV/AIDS	B20 B21 B22 B24
**Outcomes**	
Major In hospital bleed	MINAP variable: Any bleed with a drop in haemoglobin of ≥3 g/dL or a bleed related to intracranial or retroperitoneal haemorrhage (time to event not available)
Minor In hospital bleed	MINAP variable: Any bleed with a fall of < 3 g/dL of haemoglobin (time to event not available)
In hospital reinfarction	MINAP variable: The presence of clinical symptoms defined in-hospital reinfarction, new electrocardiographic changes and elevation of cardiac necrosis biomarkers beyond the upper normal limit or ≥50% of the last recorded value (time to event not available)
Major Bleeding<br><br>Neurological Haemorrhage<br><br>Gastrointestinal Bleed<br><br><br><br>Ruptured Aortic Aneurysm	 <br>I60 I61 I62 S064 S065 S066<br><br>I850 I983 K226 K250 K252 K254 K256 K260 K262 K264 K266 K270 K272 K274 K276 K280 K282 K284 K286 K290 K625 K661 K762 K920 K921 K922 I848 I844 I841<br><br>I713 I715 I711 I718
Minor Bleed	R31X R58X H113 H356 H431 H450 H922 J942 M250 N939 N950 R040 R041 R042 R048 R049 N421 N021 N022 N023 N025 N028 I230 I312 S260 N836 N857 N897 N930 N939 N938 N920 N921 N924
Ischaemic Stroke	I630 I631 I632 I633 I634 I635 I636 I638 I639
Reinfarction Admission	I210 I211 I212 I213 I214 I219 I220 I221 I228 I229
Heart Failure Admission	I110 I130 I132 I500 I501 I502 I503 I504 I508 I509

Supplementary Table 2: Multiple imputation model specification

Variable	Variable type	Imputation method	% Missing
Cardiac arrest	Binary	Logistic regression	3.4
Revascularization (PCI or CABG surgery)	Binary	Logistic regression	22.5
Hypercholesterolemia	Binary	Logistic regression	9.6
Dual antiplatelets	Binary	Logistic regression	13.4
Fondaparinux or LMWH	Binary	Logistic regression	16.9
Unfractionated heparin	Binary	Logistic regression	18.3
Warfarin	Binary	Logistic regression	18.3
Glycoprotein IIb/IIIa inhibtiors	Binary	Logistic regression	16.6
ACE inhibitor or ARB	Binary	Logistic regression	14.8
Beta-blocker	Binary	Logistic regression	11.0
High dose statin	Binary	Logistic regression	12.9
Mineralocorticoid receptor antagonist	Binary	Logistic regression	46.4
Smoking status	Categorical	Multinomial logistic regression	7.6
Elevated cardiac necrosis biomarkers	Binary	Logistic regression	5.3
Family history of coronary artery disease	Binary	Logistic regression	25.6
Ethnicity	Categorical	Multinomial logistic regression	50.1
Rehabilitation referral on discharge*	Binary	Logistic regression	10.8
Ejection fraction	Categorical	Ordered logistic regression	56.1
Killip class	Categorical	Ordered logistic regression	40.1
Creatinine	Continuous	Linear regression	21.8
Age	Continuous	Predictor variable only	0
Sex	Binary	Predictor variable only	0
Frailty category (SCARF index)	Categorical	Predictor variable only	0
Year of admission	Continuous	Predictor variable only	0
Hospital ID	Continuous	Predictor variable only	0
Final diagnosis (NSTEMI or STEMI)	Binary	Predictor variable only	0
1-year all-cause mortality censoring indicator*	Binary	Predictor variable only	0
Nelson-Aalen estimate for all-cause mortality*	Continuous	Predictor variable only	0
1-year cardiovascular mortality censoring indicator*	Binary	Predictor variable only	0
Nelson-Aalen estimate for cardiovascular mortality*	Continuous	Predictor variable only	0
1-year MACE censoring indicator*	Binary	Predictor variable only	0
Nelson-Aalen estimate for MACE*	Continuous	Predictor variable only	0
1-year heart failure censoring indicator*	Binary	Predictor variable only	0
Nelson-Aalen estimate for heart failure*	Continuous	Predictor variable only	0
1-year reinfarction censoring indicator*	Binary	Predictor variable only	0
Nelson-Aalen estimate for reinfarction*	Continuous	Predictor variable only	0
1-year major bleeding censoring indicator*	Binary	Predictor variable only	0
Nelson-Aalen estimate for major bleeding*	Continuous	Predictor variable only	0
1-year minor bleeding censoring indicator*	Binary	Predictor variable only	0

Nelson-Aalen estimate for minor bleeding*	Continuous	Predictor variable only	0
In-hospital death**	Binary	Predictor variable only	0
30-day all-cause mortality**	Binary	Predictor variable only	0
30-day cardiovascular mortality**	Binary	Predictor variable only	0
30-day MACE**	Binary	Predictor variable only	0
30-day heart failure**	Binary	Predictor variable only	0
30-day reinfarction**	Binary	Predictor variable only	0
30-day major bleeding**	Binary	Predictor variable only	0
30-day minor bleeding**	Binary	Predictor variable only	0
*1-year analysis only, **30-day analysis only			

Supplementary Figure 2: Summary of the relationship between mean SCARF index and age in males and females with AMI

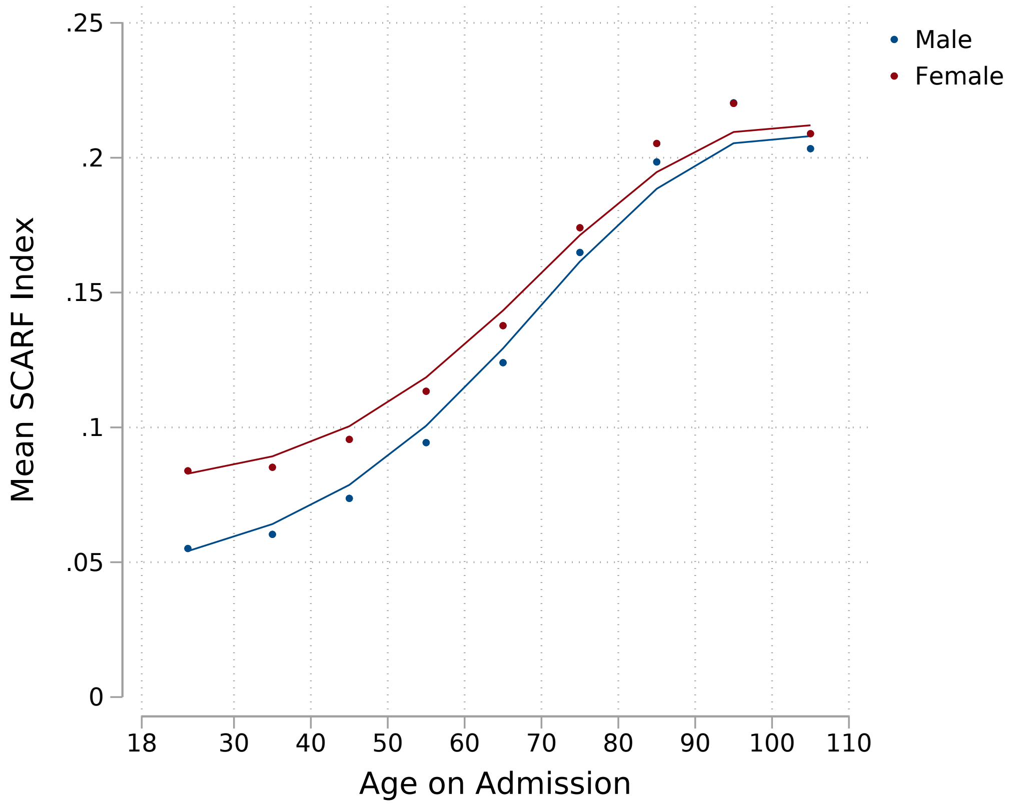

Supplementary Figure 3: Temporal trends in levels of frailty stratified by sex in patients with AMI between 2005-2019

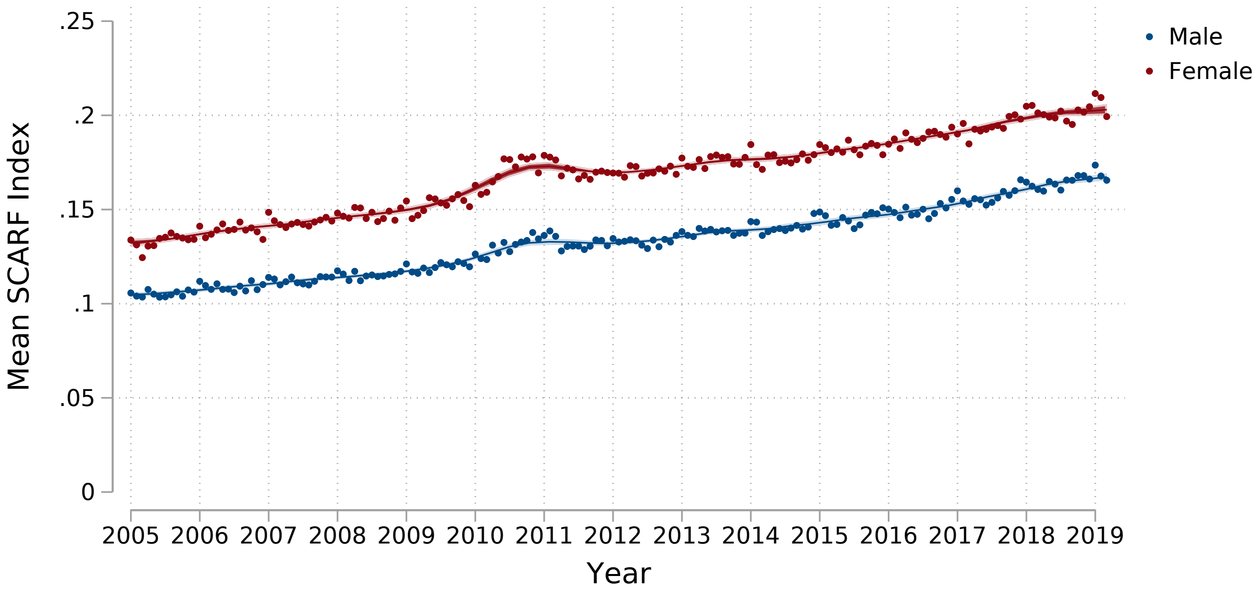

Supplementary Figure 4: Comparison of adherence to ESC quality of care indicators between males and females, stratified by frailty status

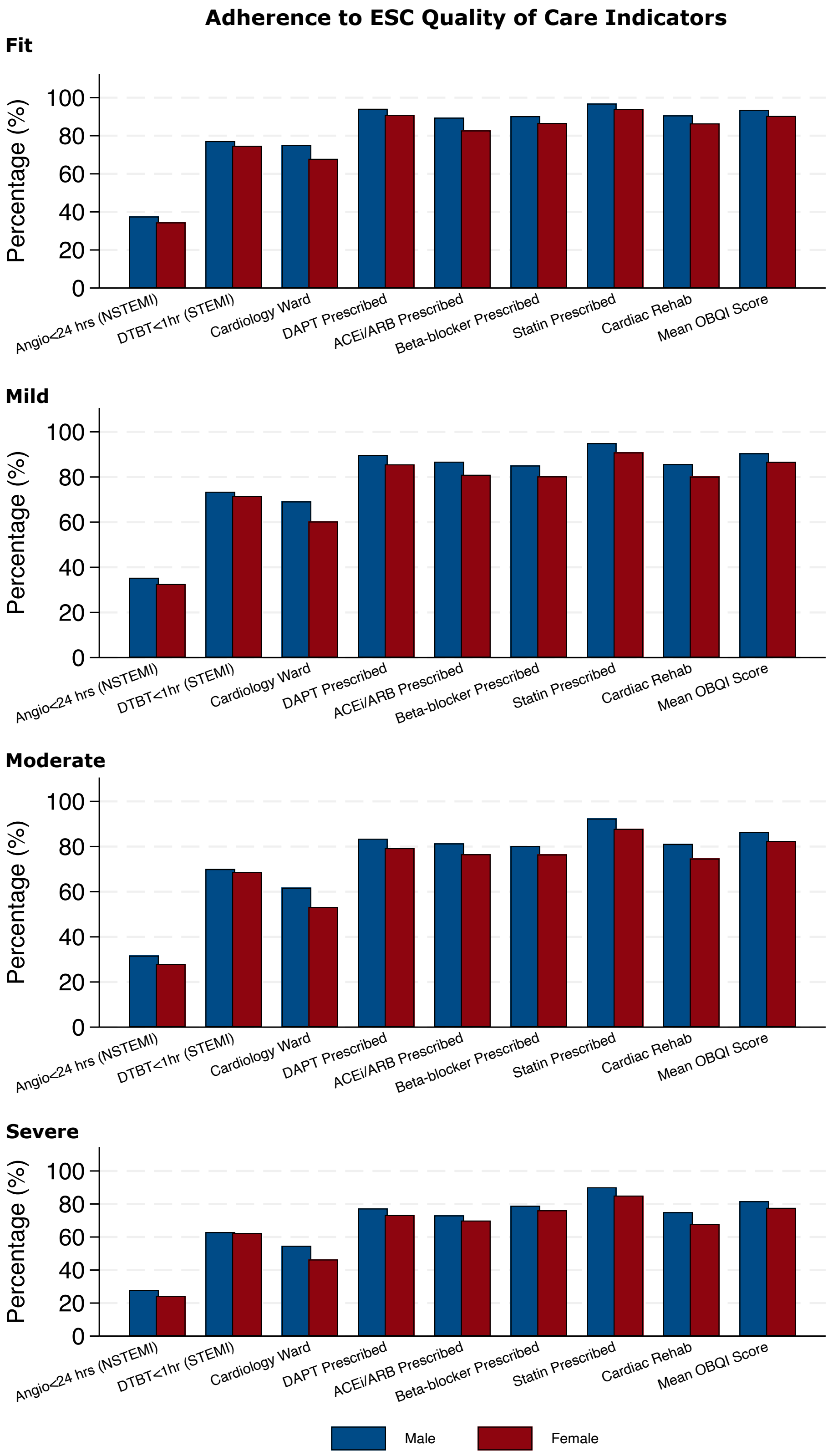

Supplementary Figure 5: Absolute risk difference in outcomes between males and females across frailty strata

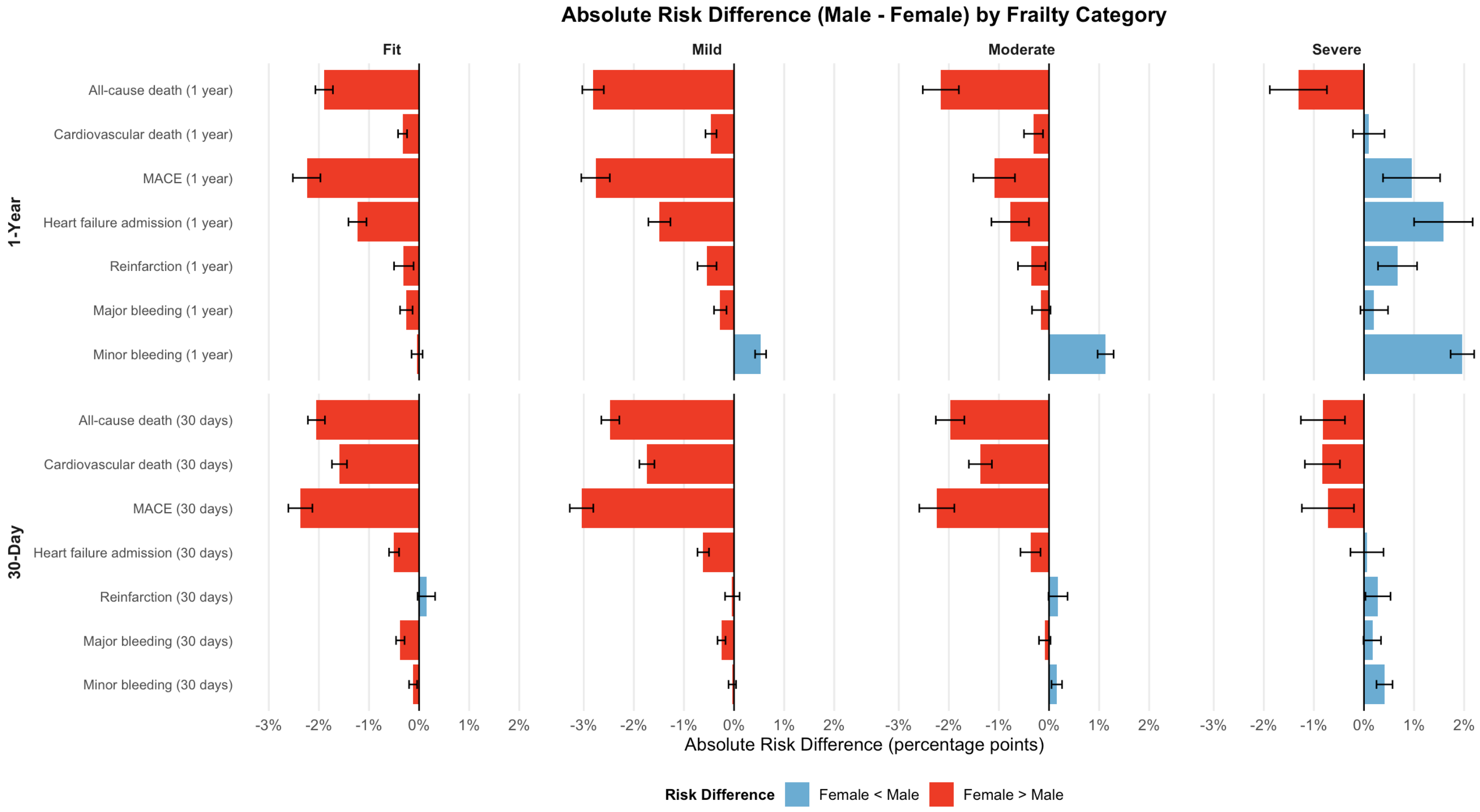

Supplementary Table 3: Absolute risk (AR) and absolute risk difference (ARD) in outcomes between males and females across frailty strata

Frailty Category	Fit					Mild					Moderate					Severe				
	AR Male	AR Female	ARD	lower	upper	AR Male	AR Female	ARD	lower	upper	AR Male	AR Female	ARD	lower	upper	AR Male	AR Female	ARD	lower	upper
**Outcome**																				
**All-cause death (30 days)**	2.5	4.5	-2.05	-2.22	-1.88	5.4	7.9	-2.47	-2.65	-2.29	10.4	12.3	-1.97	-2.26	-1.69	17.3	18.1	-0.82	-1.26	-0.38
**Cardiovascular death (30 days)**	1.9	3.5	-1.59	-1.74	-1.44	3.6	5.4	-1.74	-1.89	-1.59	6.5	7.8	-1.37	-1.60	-1.14	9.9	10.7	-0.83	-1.18	-0.48
**MACE (30 days)**	6.5	8.8	-2.37	-2.61	-2.13	10.5	13.5	-3.04	-3.28	-2.81	17.8	20.1	-2.24	-2.59	-1.89	27.5	28.2	-0.72	-1.24	-0.20
**Reinfarction (30 days)**	4.4	4.2	0.15	-0.03	0.32	4.6	4.6	-0.04	-0.18	0.11	5.1	5.0	0.18	-0.01	0.37	5.2	4.9	0.28	0.03	0.53
**Heart failure admission (30 days)**	1.0	1.5	-0.50	-0.60	-0.40	2.4	3.0	-0.62	-0.73	-0.50	5.3	5.6	-0.37	-0.57	-0.17	8.9	8.9	0.06	-0.27	0.39
**Major bleeding (30 days)**	0.7	1.0	-0.38	-0.46	-0.29	1.1	1.4	-0.25	-0.33	-0.17	1.7	1.8	-0.08	-0.20	0.03	2.4	2.3	0.17	-0.01	0.34
**Minor bleeding (30 days)**	0.7	0.8	-0.12	-0.20	-0.04	1.1	1.1	-0.03	-0.11	0.04	1.5	1.4	0.16	0.05	0.26	2.2	1.8	0.41	0.25	0.57
**All-cause death (1 year)***	2.7	4.6	-1.90	-2.07	-1.72	7.6	10.4	-2.82	-3.03	-2.60	16.9	19.0	-2.16	-2.52	-1.80	29.0	30.3	-1.31	-1.88	-0.74
**Cardiovascular death (1 year)***	0.8	1.1	-0.33	-0.42	-0.24	2.0	2.5	-0.46	-0.57	-0.35	4.1	4.4	-0.31	-0.50	-0.12	6.8	6.7	0.10	-0.22	0.41
**MACE (1 year)***	10.0	12.2	-2.24	-2.52	-1.97	18.4	21.2	-2.76	-3.05	-2.48	31.9	33.0	-1.09	-1.51	-0.68	45.1	44.2	0.95	0.38	1.52
**Reinfarction admission (1 year)***	5.1	5.4	-0.31	-0.50	-0.11	6.8	7.3	-0.54	-0.73	-0.35	9.4	9.8	-0.35	-0.62	-0.07	11.3	10.7	0.67	0.28	1.06
**Heart failure admission (1 year)***	3.5	4.7	-1.23	-1.41	-1.05	9.1	10.5	-1.49	-1.71	-1.27	19.6	20.4	-0.77	-1.15	-0.40	33.3	31.7	1.59	1.00	2.17
**Major bleeding (1 year)***	1.8	2.1	-0.26	-0.38	-0.13	2.7	3.0	-0.28	-0.40	-0.15	4.0	4.2	-0.16	-0.34	0.03	5.3	5.0	0.20	-0.07	0.48
**Minor bleeding (1 year)***	1.6	1.6	-0.04	-0.15	0.07	2.6	2.0	0.53	0.42	0.64	3.7	2.5	1.13	0.97	1.29	4.9	2.9	1.96	1.73	2.20

All numbers are expressed as percentages

*In-hospital events excluded

Lower and upper columns represent 95% confidence interval limits

Supplementary Table 4: Adjusted 1-Year outcomes comparing frailty cateogries between males and females

	Male		Female		
**Outcome**	**SCARF Index Category**	**Adjusted Hazard Ratio (95% CI)**	**Adjusted Hazard Ratio (95% CI)**	**Relative Hazard Ratio***	**P value**
**All-Cause Death**	Fit	1 (ref)	1 (ref)		
	Mild	1.72 (1.65-1.78)	1.50 (1.43-1.57)	1.18 (1.12-1.24)	<0.001
	Moderate	2.49 (2.39-2.59)	2.04 (1.94-2.16)	1.28 (1.22-1.34)	<0.001
	Severe	3.19 (3.04-3.34)	2.66 (2.51-2.82)	1.26 (1.19-1.32)	<0.001
**Cardiovascular Death**	Fit	1 (ref)	1 (ref)		
	Mild	1.68 (1.58-1.78)	1.51 (1.38-1.65)	1.10 (1.00-1.22)	0.057
	Moderate	2.35 (2.20-2.50)	2.04 (1.86-2.25)	1.13 (1.02-1.25)	0.014
	Severe	2.96 (2.74-3.19)	2.54 (2.29-2.83)	1.12 (1.02-1.24)	0.023
**MACE**	Fit	1 (ref)	1 (ref)		
	Mild	1.45 (1.41-1.50)	1.40 (1.36-1.44)	1.04 (1.01-1.08)	0.006
	Moderate	2.05 (1.98-2.12)	1.91 (1.84-1.98)	1.08 (1.05-1.12)	<0.001
	Severe	2.62 (2.51-2.74)	2.41 (2.30-2.52)	1.10 (1.06-1.13)	<0.001
**Heart Failure Readmission**	Fit	1 (ref)	1 (ref)		
	Mild	1.84 (1.76-1.93)	1.65 (1.57-1.74)	1.16 (1.06-1.27)	<0.001
	Moderate	2.88 (2.72-3.02)	2.42 (2.42-2.70)	1.25 (1.14-1.37)	<0.001
	Severe	3.91 (3.66-4.17)	3.37 (3.16-3.61)	1.28 (1.16-1.41)	<0.001
**Reinfarction**	Fit	1 (ref)	1 (ref)		
	Mild	1.21 (1.17-1.26)	1.23 (1.17-1.30)	0.97 (0.92-1.02)	0.416
	Moderate	1.52 (1.44-1.59)	1.55 (1.46-1.64)	1.00 (0.94-1.06)	0.917
	Severe	1.70 (1.60-1.81)	1.65 (1.54-1.76)	1.00 (0.92-1.09)	0.967
**Major Bleed**	Fit	1 (ref)	1 (ref)		
	Mild	1.26 (1.21-1.32)	1.27 (1.19-1.35)	1.03 (0.95-1.11)	0.479
	Moderate	1.57 (1.50-1.65)	1.59 (1.49-1.71)	1.04 (0.96-1.13)	0.292
	Severe	1.86 (1.75-1.98)	1.82 (1.68-1.97)	1.09 (1.00-1.19)	0.043
**Minor Bleed**	Fit	1 (ref)	1 (ref)		
	Mild	1.25 (1.19-1.31)	1.26 (1.16-1.35)	1.28 (1.17-1.39)	<0.001
	Moderate	1.46 (1.38-1.53)	1.56 (1.44-1.70)	1.39 (1.28-1.52)	<0.001
	Severe	1.69 (1.59-1.80)	1.77 (1.61-1.95)	1.56 (1.41-1.72)	<0.001

*Reference category is female

Supplementary Table 5: Predicted probabilities and adjusted absolute differences in outcomes between males and females across frailty strata

	Fit			Mild			Moderate			Severe		
	**Predicted Probability (95% CI)**		**Adjusted Absolute Risk Difference (95% CI)**	**Predicted Probability (95% CI)**		**Adjusted Absolute Risk Difference (95% CI)**	**Predicted Probability (95% CI)**		**Adjusted Absolute Risk Difference (95% CI)**	**Predicted Probability (95% CI)**		**Adjusted Absolute Risk Difference (95% CI)**
**Outcome**	Male	Female		Male	Female		Male	Female		Male	Female	
**All-cause death (30 days)**	1.29% (1.21% - 1.36%)	1.40% (1.26% - 1.54%)	-0.11% (-0.27% - 0.04%)	1.86% (1.77% - 1.94%)	1.75% (1.65% - 1.85%)	0.10% (-0.03% - 0.24%)	2.27% (2.16% - 2.38%)	2.05% (1.93% - 2.18%)	0.22% (0.05% - 0.38%)	2.50% (2.35% - 2.66%)	2.27% (2.11% - 2.43%)	0.23% (0.01% - 0.45%)
**Cardiovascular death (30 days)**	0.71% (0.66% - 0.77%)	0.72% (0.63% - 0.81%)	-0.01% (-0.12% - 0.10%)	0.95% (0.90% - 1.01%)	0.86% (0.79% - 0.93%)	0.09% (0.01% - 0.18%)	1.09% (1.02% - 1.17%)	1.02% (0.94% - 1.09%)	0.08% (-0.03% - 0.18%)	1.14% (1.05% - 1.24%)	1.08% (0.98% - 1.18%)	0.06% (-0.07% - 0.20%)
**MACE (30 days)**	6.46% (6.10% - 6.82%)	6.74% (6.42% - 7.06%)	-0.28% (-0.76% - 0.20%)	7.58% (7.27% - 7.89%)	7.99% (7.65% - 8.33%)	-0.41% (-0.87% - 0.06%)	9.62% (9.25% - 9.99%)	9.77% (9.42% - 10.12%)	-0.15% (-0.66% - 0.36%)	11.77% (11.30% - 12.25%)	11.80% (11.32% - 12.27%)	-0.02% (-0.70% - 0.65%)
**Heart failure admission (30 days)**	1.56% (1.44% - 1.68%)	2.17% (2.01% - 2.33%)	-0.61% (-0.81% - -0.41%)	2.86% (2.73% - 2.98%)	3.36% (3.20% - 3.53%)	-0.51% (-0.71% - -0.30%)	4.55% (4.35% - 4.74%)	4.91% (4.70% - 5.12%)	-0.37% (-0.65% - -0.08%)	6.23% (5.95% - 6.51%)	6.57% (6.29% - 6.85%)	-0.34% (-0.74% - 0.05%)
**Reinfarction (30 days)**	4.44% (4.09% - 4.80%)	4.29% (3.97% - 4.62%)	0.15% (-0.33% - 0.63%)	4.84% (4.49% - 5.19%)	4.79% (4.46% - 5.13%)	0.05% (-0.44% - 0.53%)	5.44% (5.09% - 5.80%)	5.25% (4.90% - 5.60%)	0.19% (-0.31% - 0.69%)	5.58% (5.20% - 5.96%)	5.35% (4.94% - 5.75%)	0.23% (-0.32% - 0.79%)
**Major bleeding (30 days)**	0.77% (0.69% - 0.84%)	1.08% (0.96% - 1.19%)	-0.31% (-0.45% - -0.17%)	1.11% (1.01% - 1.21%)	1.22% (1.11% - 1.34%)	-0.11% (-0.27% - 0.04%)	1.52% (1.36% - 1.68%)	1.47% (1.30% - 1.65%)	0.05% (-0.19% - 0.29%)	1.85% (1.62% - 2.08%)	1.70% (1.48% - 1.91%)	0.15% (-0.16% - 0.46%)
**Minor bleeding (30 days)**	0.84% (0.69% - 0.99%)	0.89% (0.72% - 1.06%)	-0.05% (-0.28% - 0.18%)	1.10% (0.91% - 1.29%)	1.01% (0.81% - 1.21%)	0.09% (-0.18% - 0.37%)	1.38% (1.14% - 1.62%)	1.12% (0.87% - 1.37%)	0.26% (-0.09% - 0.61%)	1.82% (1.49% - 2.15%)	1.34% (1.03% - 1.65%)	0.48% (0.02% - 0.93%)
**All-cause death (1 year)***	6.42% (6.19% - 6.65%)	7.08% (6.77% - 7.38%)	-0.66% (-1.04% - -0.27%)	10.12% (9.89% - 10.36%)	9.85% (9.57% - 10.13%)	0.28% (-0.09% - 0.64%)	14.04% (13.75% - 14.34%)	12.93% (12.62% - 13.24%)	1.11% (0.69% - 1.54%)	17.78% (17.37% - 18.20%)	16.59% (16.16% - 17.03%)	1.19% (0.58% - 1.79%)
**Cardiovascular death (1 year)***	11.75% (11.28% - 12.22%)	12.44% (12.02% - 12.86%)	-0.69% (-1.32% - -0.06%)	15.93% (15.53% - 16.33%)	16.53% (16.08% - 16.97%)	-0.59% (-1.19% - 0.00%)	21.74% (21.23% - 22.25%)	22.04% (21.53% - 22.54%)	-0.30% (-1.02% - 0.42%)	27.37% (26.61% - 28.13%)	26.76% (26.02% - 27.49%)	0.61% (-0.45% - 1.67%)
**MACE (1 year)***	16.88% (16.36% - 17.40%)	17.43% (16.93% - 17.92%)	-0.55% (-1.27% - 0.17%)	22.33% (21.89% - 22.77%)	22.56% (22.05% - 23.06%)	-0.23% (-0.90% - 0.44%)	29.71% (29.15% - 30.26%)	29.28% (28.74% - 29.82%)	0.43% (-0.35% - 1.20%)	37.87% (37.01% - 38.73%)	36.65% (35.85% - 37.45%)	1.22% (0.04% - 2.39%)
**Heart Failure admission (1 year)***	5.87% (5.56% - 6.19%)	7.00% (6.65% - 7.34%)	-1.13% (-1.59% - -0.66%)	10.27% (9.95% - 10.59%)	10.89% (10.56% - 11.22%)	-0.62% (-1.08% - -0.16%)	15.42% (14.99% - 15.84%)	15.89% (15.46% - 16.31%)	-0.47% (-1.07% - 0.13%)	20.43% (19.78% - 21.09%)	20.28% (19.62% - 20.93%)	0.15% (-0.77% - 1.08%)

**Reinfarction admission (1 year)***	6.04% (5.68% - 6.41%)	5.95% (5.60% - 6.31%)	0.09% (-0.42% - 0.60%)	7.34% (7.01% - 7.67%)	7.36% (6.99% - 7.72%)	-0.02% (-0.51% - 0.48%)	9.13% (8.74% - 9.51%)	9.09% (8.68% - 9.51%)	0.03% (-0.53% - 0.60%)	9.97% (9.54% - 10.39%)	9.40% (8.95% - 9.85%)	0.57% (-0.05% - 1.19%)
**Major bleeding (1 year)***	2.31% (2.21% - 2.42%)	2.31% (2.17% - 2.46%)	0.00% (-0.18% - 0.18%)	2.95% (2.85% - 3.04%)	2.88% (2.77% - 2.99%)	0.06% (-0.08% - 0.21%)	3.70% (3.57% - 3.83%)	3.49% (3.35% - 3.62%)	0.21% (0.03% - 0.40%)	4.07% (3.89% - 4.26%)	3.78% (3.59% - 3.98%)	0.29% (0.02% - 0.56%)
**Minor bleeding (1 year)***	2.01% (1.92% - 2.09%)	1.83% (1.70% - 1.96%)	0.18% (0.02% - 0.33%)	2.74% (2.65% - 2.84%)	1.94% (1.85% - 2.03%)	0.81% (0.67% - 0.94%)	3.31% (3.17% - 3.45%)	2.12% (2.01% - 2.24%)	1.19% (1.01% - 1.37%)	3.76% (3.57% - 3.96%)	2.21% (2.06% - 2.36%)	1.55% (1.30% - 1.80%)
*In-hospital events excluded												

Supplementary Table 6: Adjusted 30-day outcomes comparing frailty cateogries between males and females

	Male		Female		
**Outcome**	**SCARF Index Category**	**Adjusted Odds Ratio (95% CI)**	**Adjusted Odds Ratio (95% CI)**	**Relative Odds Ratio***	**P value**
**All-Cause Death**	Fit	1 (ref)	1 (ref)		
	Mild	1.44 (1.34-1.54)	1.29 (1.16-1.44)	1.13 (1.03-1.19)	0.003
	Moderate	1.81 (1.68-1.96)	1.56 (1.38-1.75)	1.19 (1.12-1.27)	<0.001
	Severe	2.09 (1.91-2.28)	1.77 (1.56-2.01)	1.23 (1.15-1.31)	<0.001
**Cardiovascular Death**	Fit	1 (ref)	1 (ref)		
	Mild	1.32 (1.22-1.44)	1.22 (1.05-1.41)	1.09 (1.02-1.17)	0.018
	Moderate	1.55 (1.40-1.72)	1.47 (1.26-1.72)	1.16 (1.08-1.25)	<0.001
	Severe	1.68 (1.49-1.89)	1.62 (1.37-1.90)	1.16 (1.08-1.25)	<0.001
**MACE**	Fit	1 (ref)	1 (ref)		
	Mild	1.20 (1.16-1.24)	1.21 (1.16-1.27)	0.99 (0.95-1.03)	0.587
	Moderate	1.58 (1.52-1.64)	1.53 (1.46-1.61)	1.03 (0.99-1.08)	0.137
	Severe	2.01 (1.92-2.12)	1.99 (1.80-2.02)	1.05 (1.00-1.10)	0.022
**Heart Failure Readmission**	Fit	1 (ref)	1 (ref)		
	Mild	1.81 (1.71-1.93)	1.60 (1.48-1.72)	1.17 (1.07-1.28)	0.001
	Moderate	2.92 (2.71-3.15)	2.43 (2.24-2.64)	1.26 (1.16-1.38)	<0.001
	Severe	4.02 (3.69-4.39)	3.32 (3.03-3.64)	1.29 (1.18-1.41)	<0.001
**Reinfarction**	Fit	1 (ref)	1 (ref)		
	Mild	1.10 (1.05-1.16)	1.15 (1.08-1.22)	0.97 (0.92-1.02)	0.234
	Moderate	1.26 (1.19-1.34)	1.30 (1.21-1.40)	1.00 (0.94-1.06)	0.94
	Severe	1.30 (1.21-1.39)	1.36 (1.25-1.48)	1.00 (0.94-1.07)	0.927
**Major Bleed**	Fit	1 (ref)	1 (ref)		
	Mild	1.39 (1.29-1.51)	1.22 (1.11-1.34)	1.28 (1.15-1.44)	<0.001
	Moderate	1.79 (1.62-1.97)	1.53 (1.37-1.72)	1.45 (1.29-1.62)	<0.001
	Severe	2.15 (1.91-2.42)	1.76 (1.55-2.01)	1.57 (1.40-1.77)	<0.001
**Minor Bleed**	Fit	1 (ref)	1 (ref)		
	Mild	1.18 (1.09-1.29)	1.26 (1.13-1.40)	1.13 (1.00-1.28)	0.042
	Moderate	1.36 (1.24-1.50)	1.51 (1.33-1.71)	1.26 (1.12-1.43)	<0.001
	Severe	1.69 (1.49-1.89)	1.80 (1.59-2.04)	1.34 (1.18-1.53)	<0.001

*Reference category is female

Supplementary Table 7: Adjusted 1-year Outcomes Across Frailty Categories Between Men and Women in STEMI and NSTEMI

		STEMI				NSTEMI			
		Male	Female			Male	Female		
	SCARF Index Category	1-year Adjusted Hazard Ratio (95% CI)		Relative Hazard Ratio (95% CI)	P-interaction	1-year Adjusted Hazard Ratio (95% CI)		Relative Hazard Ratio (95% CI)	P-interaction
**Outcome**									
**All-Cause Death**	Fit	1 (ref)	1 (ref)			1 (ref)	1 (ref)		
	Mild	1.61 (1.53-1.70)	1.45 (1.35-1.56)	1.15 (1.06-1.25)	0.001	1.72 (1.65-1.79)	1.50 (1.42-1.58)	1.15 (1.06-1.25)	0.001
	Moderate	2.41 (2.27-2.56)	2.00 (1.86-2.16)	1.29 (1.18-1.40)	<0.001	2.44 (2.34-2.55)	2.01 (1.91-2.12)	1.29 (1.18-1.40)	<0.001
	Severe	3.26 (3.05-3.50)	2.67 (2.46-2.90)	1.32 (1.21-1.45)	<0.001	3.12 (2.99-3.26)	2.61 (2.48-2.75)	1.32 (1.21-1.45)	<0.001
**Cardiovascular Death**	Fit	1 (ref)	1 (ref)			1 (ref)	1 (ref)		
	Mild	1.51 (1.39-1.65)	1.46 (1.29-1.66)	1.04 (0.90-1.21)	0.57	1.78 (1.63-1.93)	1.50 (1.35-1.68)	1.16 (1.01-1.33)	0.039
	Moderate	2.10 (1.90-2.31)	1.91 (1.66-2.18)	1.13 (0.97-1.32)	0.115	2.45 (2.25-2.67)	2.03 (1.82-2.27)	1.15 (1.01-1.32)	0.038
	Severe	2.63 (2.34-2.96)	2.45 (2.11-2.84)	1.10 (0.94-1.30)	0.237	3.14 (2.86-3.45)	2.53 (2.25-2.84)	1.15 (1.01-1.32)	0.041
**MACE**	Fit	1 (ref)	1 (ref)			1 (ref)	1 (ref)		
	Mild	1.39 (1.35-1.43)	1.29 (1.24-1.35)	1.07 (1.02-1.13)	0.004	1.52 (1.48-1.55)	1.48 (1.44-1.53)	1.02 (0.98-1.07)	0.266
	Moderate	1.90 (1.84-1.97)	1.71 (1.63-1.79)	1.11 (1.06-1.17)	<0.001	2.15 (2.09-2.20)	2.03 (1.96-2.10)	1.06 (1.01-1.10)	0.008
	Severe	2.45 (2.35-2.55)	2.17 (2.05-2.29)	1.13 (1.07-1.20)	<0.001	2.75 (2.67-2.82)	2.55 (2.46-2.65)	1.07 (1.02-1.11)	0.002
**Heart Failure Readmission**	Fit	1 (ref)	1 (ref)			1 (ref)	1 (ref)		
	Mild	1.68 (1.61-1.75)	1.43 (1.34-1.52)	1.19 (1.11-1.28)	<0.001	2.02 (1.94-2.10)	1.86 (1.76-1.96)	1.09 (1.04-1.15)	<0.001
	Moderate	2.36 (2.25-2.48)	2.04 (1.91-2.18)	1.19 (1.10-1.28)	<0.001	3.27 (3.14-3.41)	2.92 (2.76-3.09)	1.13 (1.07-1.19)	<0.001
	Severe	3.07 (2.89-3.27)	2.60 (2.41-2.80)	1.24 (1.14-1.35)	<0.001	4.47 (4.28-4.67)	3.89 (3.68-4.12)	1.16 (1.09-1.23)	<0.001
**Reinfarction**	Fit	1 (ref)	1 (ref)			1 (ref)	1 (ref)		
	Mild	1.21 (1.16-1.26)	1.25 (1.21-1.30)	1.05 (0.97-1.13)	0.201	1.15 (1.08-1.23)	1.32 (1.25-1.40)	0.94 (0.89-1.00)	0.066
	Moderate	1.46 (1.38-1.55)	1.57 (1.51-1.64)	1.06 (0.97-1.16)	0.188	1.39 (1.28-1.50)	1.67 (1.58-1.77)	0.94 (0.89-1.00)	0.063
	Severe	1.71 (1.58-1.85)	1.73 (1.65-1.81)	1.03 (0.93-1.15)	0.555	1.68 (1.53-1.86)	1.71 (1.61-1.83)	1.01 (0.94-1.08)	0.864
**Major Bleed**	Fit	1 (ref)	1 (ref)			1 (ref)	1 (ref)		
	Mild	1.18 (1.10-1.26)	1.22 (1.10-1.36)	0.98 (0.87-1.11)	0.775	1.31 (1.24-1.39)	1.28 (1.18-1.39)	1.06 (0.96-1.17)	0.233
	Moderate	1.41 (1.30-1.54)	1.48 (1.31-1.67)	0.98 (0.86-1.13)	0.804	1.66 (1.55-1.76)	1.62 (1.48-1.76)	1.08 (0.98-1.19)	0.119
	Severe	1.76 (1.57-1.98)	1.96 (1.69-2.26)	0.94 (0.80-1.10)	0.458	1.94 (1.80-2.09)	1.79 (1.62-1.96)	1.15 (1.04-1.27)	0.009

**Minor Bleed**	Fit	1 (ref)	1 (ref)			1 (ref)	1 (ref)		
	Mild	1.17 (1.09-1.25)	1.34 (1.18-1.52)	1.09 (0.95-1.25)	0.231	1.30 (1.22-1.38)	1.20 (1.09-1.32)	1.40 (1.26-1.56)	<0.001
	Moderate	1.29 (1.18-1.41)	1.72 (1.48-1.99)	1.11 (0.95-1.30)	0.192	1.53 (1.43-1.64)	1.48 (1.33-1.64)	1.54 (1.38-1.72)	<0.001
	Severe	1.65 (1.46-1.86)	1.72 (1.42-2.08)	1.63 (1.34-1.98)	<0.001	1.75 (1.62-1.89)	1.73 (1.54-1.94)	1.60 (1.42-1.80)	<0.001

Supplementary Table 8: Adjusted 30-day outcomes comparing sex differences across frailty cateogries stratified by AMI subtype

		STEMI				NSTEMI			
		**Male**	**Female**			**Male**	**Female**		
	**SCARF Index Category**	**30 day Adjusted Odds Ratio (95% CI)**		**Relative Odds Ratio (95% CI)**	**P value**	**30 day Adjusted Odds Ratio (95% CI)**		**Relative Odds Ratio (95% CI)**	**P value**
Outcome									
**All-Cause Death**	Fit	1 (ref)	1 (ref)			1 (ref)	1 (ref)		
	Mild	1.20 (1.14-1.26)	1.14 (1.07-1.23)	1.05 (0.96-1.14)	0.265	1.37 (1.29-1.46)	1.28 (1.18-1.38)	1.10 (1.00-1.21)	0.059
	Moderate	1.46 (1.37-1.55)	1.23 (1.14-1.33)	1.19 (1.09-1.31)	<0.001	1.73 (1.62-1.84)	1.59 (1.47-1.72)	1.11 (1.01-1.22)	0.034
	Severe	1.75 (1.63-1.89)	1.36 (1.25-1.48)	1.28 (1.15-1.41)	<0.001	2.28 (2.12-2.45)	2.03 (1.87-2.21)	1.13 (1.02-1.24)	0.015
**Cardiovascular Death**	Fit	1 (ref)	1 (ref)			1 (ref)	1 (ref)		
	Mild	1.09 (1.03-1.16)	1.01 (0.94-1.09)	1.08 (0.98-1.18)	0.104	1.14 (1.05-1.23)	1.11 (1.01-1.22)	1.02 (0.90-1.16)	0.719
	Moderate	1.21 (1.13-1.29)	1.00 (0.92-1.08)	1.20 (1.09-1.32)	<0.001	1.30 (1.20-1.41)	1.27 (1.15-1.40)	1.01 (0.90-1.14)	0.871
	Severe	1.24 (1.14-1.34)	0.98 (0.89-1.08)	1.23 (1.10-1.37)	<0.001	1.51 (1.38-1.65)	1.45 (1.31-1.61)	1.00 (0.88-1.12)	0.951
**MACE**	Fit	1 (ref)	1 (ref)			1 (ref)	1 (ref)		
	Mild	1.17 (1.13-1.21)	1.14 (1.08-1.20)	0.99 (0.93-1.05)	0.731	1.15 (1.11- 1.19)	1.20 (1.14-1.26)	0.94 (0.88-1.00)	0.038
	Moderate	1.46 (1.40-1.53)	1.31 (1.23-1.39)	1.04 (0.98-1.12)	0.199	1.49 (1.43-1.55)	1.51 (1.43-1.59)	0.95 (0.90-1.01)	0.106
	Severe	1.86 (1.76-1.97)	1.54 (1.44-1.65)	1.09 (1.01-1.18)	0.032	1.93 (1.85-2.02)	1.90 (1.80-2.01)	0.96 (0.90-1.02)	0.144
**Heart Failure Readmission**	Fit	1 (ref)	1 (ref)			1 (ref)	1 (ref)		
	Mild	1.69 (1.57-1.83)	1.47 (1.32-1.64)	1.20 (1.05-1.36)	0.006	1.89 (1.74-2.04)	1.65 (1.49-1.82)	1.15 (1.01-1.30)	0.031
	Moderate	2.36 (2.17-2.58)	2.11 (1.88-2.37)	1.21 (1.06-1.38)	0.004	3.17 (2.92-3.43)	2.49 (2.25-2.76)	1.27 (1.12-1.44)	<0.001
	Severe	3.08 (2.78-3.42)	2.63 (2.31-2.99)	1.32 (1.14-1.53)	<0.001	4.37 (4.01-4.75)	3.43 (3.09-3.80)	1.26 (1.12-1.43)	<0.001
**Reinfarction**	Fit	1 (ref)	1 (ref)			1 (ref)	1 (ref)		
	Mild	1.13 (1.08-1.18)	1.13 (1.05-1.21)	1.02 (0.94-1.10)	0.67	1.04 (0.99-1.09)	1.15 (1.08-1.23)	0.92 (0.85-0.99)	0.026
	Moderate	1.29 (1.22-1.38)	1.26 (1.15-1.38)	1.06 (0.96-1.16)	0.266	1.14 (1.08-1.20)	1.25 (1.16-1.34)	0.95 (0.87-1.03)	0.18
	Severe	1.37 (1.26-1.50)	1.52 (1.36-1.70)	0.93 (0.83-1.05)	0.266	1.09 (1.03-1.17)	1.17 (1.08-1.28)	0.98 (0.90-1.07)	0.712
**Major Bleed**	Fit	1 (ref)	1 (ref)			1 (ref)	1 (ref)		
	Mild	1.52 (1.38-1.67)	1.25 (1.10-1.43)	1.28 (1.09-1.50)	0.002	1.30 (1.18-1.44)	1.21 (1.06-1.38)	1.23 (1.05-1.45)	0.01

	Moderate	1.90 (1.69-2.14)	1.60 (1.38-1.85)	1.32 (1.11-1.57)	0.001	1.67 (1.50-1.86)	1.45 (1.26-1.66)	1.43 (1.22-1.67)	<0.001
	Severe	2.30 (1.99-2.67)	1.72 (1.44-2.06)	1.54 (1.26-1.87)	<0.001	1.97 (1.74-2.22)	1.73 (1.49-2.00)	1.48 (1.26-1.74)	<0.001
**Minor Bleed**	Fit	1 (ref)	1 (ref)			1 (ref)	1 (ref)		
	Mild	1.27 (1.15-1.40)	1.29 (1.11-1.51)	1.12 (0.94-1.34)	0.211	1.19 (1.08-1.31)	1.26 (1.10-1.46)	1.11 (0.94-1.30)	0.237
	Moderate	1.50 (1.32-1.70)	1.57 (1.32-1.87)	1.22 (1.00-1.48)	0.046	1.42 (1.28-1.58)	1.51 (1.30-1.75)	1.23 (1.04-1.45)	0.015
	Severe	1.75 (1.49-2.05)	1.77 (1.43-2.18)	1.38 (1.10-1.73)	0.006	1.81 (1.61-2.04)	2.00 (1.71-2.35)	1.27 (1.07-1.50)	0.006
